# Antibiotic Resistance Genes in Salmonella Isolates from Food Production Environments and Clinical Sources in the Middle East: A Systematic Review and Meta-Analysis Revealing the Limited Comparative Genomic Evidence

**DOI:** 10.3390/life16091458

**Published:** 2026-08-31

**Authors:** Mohammad Al-Hasan, Ahmad Al-Dhumair, Hanan Al-Khalaifah, Nazima Habibi, Qadreyah Almatawah

**Affiliations:** Food Security Program, Kuwait Institute for Scientific Research, Safat, Kuwait City 13109, Kuwait; aazmi@kisr.edu.kw (A.A.-D.); hkhalifa@kisr.edu.kw (H.A.-K.); nhabibi@kisr.edu.kw (N.H.); qmutawa@kisr.edu.kw (Q.A.)

**Keywords:** *Salmonella*, antibiotic resistant genes, multiple drug resistance, ARGs, Middle East, GCC

## Abstract

This systematic review collects available evidence on genetic similarities between antibiotic resistance genes (ARGs) in *Salmonella* isolates collected from both food production environments and hospitalized patients in the Middle East (ME) region. We conducted an extensive search across PubMed, ScienceDirect, Scopus, Web of Science, and Google Scholar, following PRISMA guidelines, to cover all available literature up to May 2026. Finally, 94 studies met our inclusion criteria. Overall pooled prevalence of *Salmonella* isolates was 0.30 (95% CI: 0.23–0.36, *p* < 0.001, I^2^ = 99.92%). The prevalence of specific classes (β-lactamases [ESBLs], quinolones, chloramphenicol, aminoglycosides, and tetracyclines) was 0.49 (95% CI, 0.31 to 0.66, *p* < 0.001, I^2^ = 98.47%). Our findings further demonstrate a high diversity of classes, with β-lactamase genes (*bla*_TEM_, *bla*_CTXM_, *bla*_SHV_, *bla*_CMY_, *bla*_OXA_, and *bla*_NDM_), which were most commonly detected across the ME region in the food production environment and clinical isolates 0.48 (95% CI, 0.37 to 0.58, *p* < 0.001, I^2^ = 99.34%). Although studies have documented resistance against different classes in both food production environments and clinical *Salmonella* isolates in the ME, however, the direct comparative genomic evidence demonstrating genetic similarity and transmission between these sources is still missing. Further research is needed to investigate the application of WGS to directly compare isolates from food production environments and clinical isolates within the same geographic areas.

## 1. Introduction

*Salmonella*, a Gram-negative bacterium, remains one of the worldwide leading causes of foodborne illness, with approximately 93.8 million cases and 155,000 deaths annually [1,2]. *Salmonella* serovars are capable of infecting and colonizing a wide range of humans, animals, and their surrounding environment. These *Salmonella* serovars also represent a major zoonotic pathogen, particularly for food-producing animals serving as a reservoir for transmission in humans [3]. The situation in the Middle East (ME), especially in Gulf Cooperation Council (GCC) countries, is at high risk because this region heavily depends on imported food products, carrying *Salmonella* and cause significant public health and economic burdens [4,5]. What makes this challenge even more worrying is the emergence and spread of antibiotic resistance (AMR) in *Salmonella*, which not only complicates clinical management but also threatens food safety in the region [6,7,8]. Many other contributing factors make the ME exposed to foodborne AMR transmission. First, the hot climate poses challenges to maintaining cold chains during food transport and storage. Second, fast growth has led to changes in food consumption habits with increased dependence on imported food. In addition, differences in food safety regulations and surveillance systems in different ME countries can allow resistant strains to spread [4,5].

The farm-to-fork journey demonstrates an important pathway by which resistant *Salmonella* serovars can spread [9,10,11]. The use of antibiotics in livestock, poultry production and humans introduces a selective pressure promoting the development of AMR in *Salmonella* [12,13,14]. These resistant *Salmonella* serovars may then contaminate food products, and as end users, humans are at high risk of infection [14]. Thus, understanding the genetic association between *Salmonella* isolates from food production environments and those responsible for clinical infections has become important for applying effective One Health strategies [4,15]. Meanwhile, the increasing rate of AMR poses a significant global concern for food security and requires an integrated surveillance system [16]. One Health approaches link animal, environmental, and human systems along with antimicrobial stewardship [2]. This One Health approach is helpful in engaging *Salmonella* resistance by integrating multidisciplinary disciplines, which ensures food safety, preserves public health, and minimizes the global threat of resistance [14]. Additionally, this approach further involves the development of collaborative systems for effective monitoring of the emergence and movement of genes associated with resistance and resistant bacteria within and between biological compartments [17].

The recent developments in molecular typing techniques have improved our capacity to follow bacterial transmission. Whole Genome Sequencing (WGS) is a powerful tool providing an extraordinary solution for identifying genetic relationships between isolates [18]. In addition, researchers can apply WGS to detect and differentiate shared antibiotic resistance genes (ARGs), mobile genetic elements, and sequence types (STs) that can specify transmission events between different reservoirs with high resolution [19,20].

Even with these technological developments, our understanding of how resistant *Salmonella* transfers between food production and clinical environments in ME remains limited. A previously published review has investigated AMR prevalence in the region; however, that review did not focus on the genetic similarity between food production environments and clinical isolates [4]. This knowledge gap is challenging because without performing comparative genomic analyses, the transmission pathways and evolutionary relationships of the extra-domain isolates cannot be established. Thus, this prevents efforts for targeted interventions, such as the judicious use of antibiotics in animals and humans, which can break transmission chains. Moreover, the genetic similarity investigations between food production and clinical *Salmonella* isolates demonstrate a significant implication for public health policy. If we can show that resistant strains in food production and those causing human infections share similar ARGs, this would provide strong evidence for implementing firm control on antibiotic use in agriculture and humans. It would also justify improved surveillance programs. Therefore, this systematic review aims to address these knowledge gaps by reporting all available evidence on the genetic similarity of ARGs in *Salmonella* isolates from both food production and clinical environments in the ME regions. In this systematic review, we want to answer the following questions: (1) What is the extent and availability of genetic similarity between ARGs in *Salmonella* from the two domains? (2) Which resistance genes are most prevalent in each domain? (3) Are there shared STs that suggest transmission pathways? (4) What is the quality of evidence supporting any observed relation? By systematically evaluating the existing literature, we hope to not only summarize what we now know but also to identify significant research gaps that need to be addressed. Our findings should update both research priorities and public health interventions aimed at controlling AMR transmission through the food chain in ME countries.

## 2. Materials and Methods

### 2.1. Protocol and Registration

We developed this systematic review following the Preferred Reporting Items for Systematic Reviews and Meta-Analyses (PRISMA) 2020 guidelines [21]. The protocol was registered prospectively with PROSPERO (registration number: CRD420261384420; dated: 1 July 2026) prior to initiating the literature search. Any deviations from the original protocol were documented and reported transparently. The completed PRISMA 2020 checklist is provided in the Supplementary Table S1.

### 2.2. Eligibility Criteria

We established our inclusion and exclusion criteria using the PECOS (Population, Exposure, Comparison, Outcome, and Study design) framework [22]:

**Population: ***Salmonella* isolates from Middle Eastern (ME) countries (not limited to GCC countries), including Iran, Iraq, Jordan, Lebanon, Yemen, Egypt, and Syria.
**Exposure**: *Salmonella* isolates collected from food production environments, including farms, slaughterhouses, food processing facilities, retail food products, livestock, poultry, and imported food items, or clinical isolates from hospitalized patients.
**Comparison**: Studies comparing genetic characterizations between both food production and clinical isolates were selected, but studies reporting data from either source alone were also included to support indirect comparisons.
**Outcomes**: The primary outcomes included the prevalence of *Salmonella*, ARGs, genetic similarity of ARGs, prevalence of specific resistance genes (*bla_CTXM_*, *bla_CMY_*, *mcr*, and *tet* genes), STs, and mobile genetic elements (plasmids, integrons, and transposons). The secondary outcomes included phenotypic AMR patterns.
**Study designs:** We included observational studies, including cross-sectional, case–control, cohort, and surveillance studies, as well as outbreak investigations that apply molecular characterization methods such as Polymerase Chain Reaction (PCR), WGS, Multilocus Sequence Typing (MLST), and Pulsed-Field Gel Electrophoresis (PFGE), among others.
**Exclusion criteria**: The excluded studies (1) did not report molecular characterization or resistance gene data, (2) focused only on typhoidal *Salmonella* such as *S. typhi* and *S. paratyphi* without including non-typhoidal *Salmonella*, (3) were conducted outside ME regions, (4) were reviews, commentaries, or conference abstracts without original data, (5) were not available in the English language, and (6) did not provide sufficient methodological detail regarding sample size, eligibility criteria, molecular methods, or statistical analysis to assess methodological quality.

### 2.3. Literature Search Strategy

We conducted a comprehensive literature search across different electronic databases, including PubMed (MEDLINE), ScienceDirect, Scopus, Web of Science, and Google Scholar. The search covered all available literature from inception to May 2026. The search was restricted using database filters based on language, study settings, and geographical location. We developed a sensitive search strategy combining terms related to (1) *Salmonella*, (2) antibiotic/AMR and resistance genes, (3) food production, livestock, poultry, and clinical/hospital settings, (4) molecular methods and genetic similarity, and finally, (5) ME countries and regions.

The core search was adapted for each database’s syntax, and modified across the databases:

(“*Salmonella*” OR “*Salmonella enterica*”) AND (“antibiotic resistance” OR “antibiotic resistance bacteria” OR “antibiotic-resistant bacteria” OR “antimicrobial resistance” OR “AMR” OR “resistance gene” OR “antibiotic resistance gene” OR “ARG” OR “blaCTX-M” OR “blaCMY” OR “mcr” OR “tet gene”) AND (“food production” OR “livestock” OR “poultry” OR “farms” OR “slaughterhouse” OR “food processing” OR “retail food” OR “clinical*” OR “hospital*” OR “patient*” OR “genetic similarity” OR “whole genome sequencing” OR “WGS” OR “multilocus sequence typing” OR “sequence type” OR “molecular typing” OR “comparative genomics”) AND (“Middle East” OR “Middle East Regions” OR “Middle East countries” OR “Gulf Cooperation Council countries” OR “GCC” OR “Gulf” OR “UAE” OR “Saudi Arabia” OR “Kuwait” OR “Qatar” OR “Bahrain” OR “Oman” OR “Iran” OR “Iraq” OR “Jordan” OR “Lebanon” Or “Egypt” OR “Yemen” OR “Syria”)

We supplemented database searches by (1) screening reference lists of included studies and relevant reviews, (2) conducting forward citation searches of key articles, and (3) searching gray literature sources, including government reports and surveillance databases. Full search strategies for all databases are provided in the Supplementary Table S2.

### 2.4. Selection Process

All results were imported into EndNote X9 reference management software. All duplicate records were identified and removed using both automated and manual methods. Titles and abstracts were screened against the eligibility criteria using a standardized PRISMA flowchart. Studies marked as potentially relevant proceeded to full-text review.

**Figure 1:**
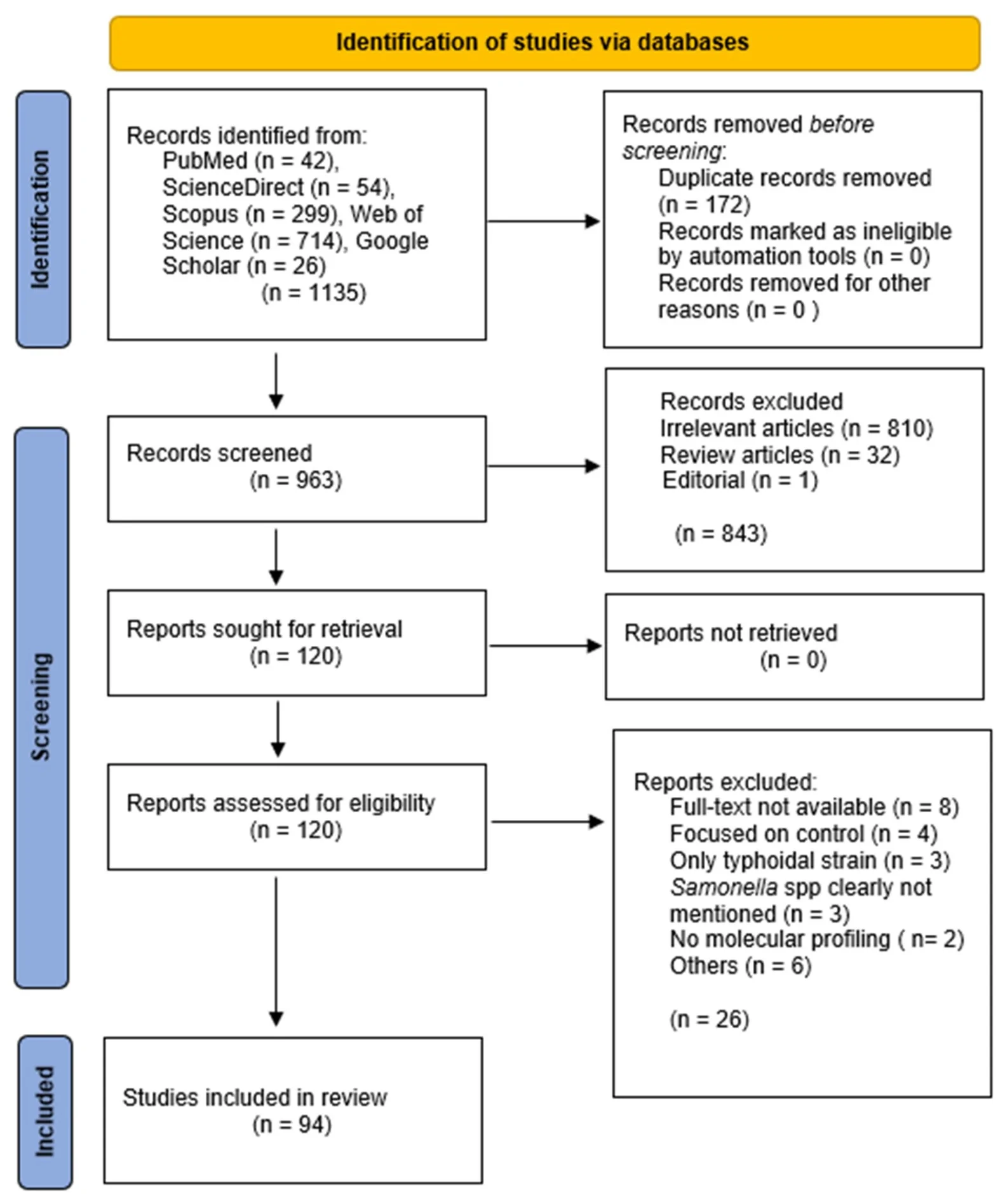
PRISMA flowchart illustrating the screening of relevant articles.

For full-text screening, we assessed each article against the detailed inclusion and exclusion criteria. We documented reasons for exclusion at the full-text stage (Figure 1). This entire process was performed by two independent reviewers, and in case of discrepancy, a third senior reviewer was consulted.

### 2.5. Data Collection Process

We developed a standardized data extraction form based on the Cochrane Collaboration guidelines [23] and piloted it on five randomly selected included studies. When studies reported data in formats that required conversion or calculation (such as percentages without raw numbers), we contacted study authors to request additional information. If the authors did not respond within four weeks, we extracted available data and noted the limitations in our analysis. We extracted the following data items from each included study:

**Study characteristics**: First author, publication year, country, study design, and sample size.
**Population characteristics**: Source of isolates (food production: specific animal species, food products, production stage), key *Salmonella* serovars, sampling method. Clinical: infection type and sampling method, prevalence of *Salmonella*.
**Molecular methods:** Molecular methods used, typing methods used (WGS, MLST, etc.).
**Resistance gene data**: Specific ARGs detected, prevalence of ARGs, resistant gene variants, prevalence of each gene, location (chromosomal vs. plasmid), and mobile genetic elements (integrons, transposons, plasmid types).
**Genetic typing results:** STs.
**Phenotypic resistance**: Resistance to antibiotics, MDR prevalence.

### 2.6. Methodological Quality Assessment

We used a modified version of the Joanna Briggs Institute (JBI) Critical Appraisal Checklist for Prevalence Studies, which was adapted for molecular epidemiology studies to assess risk of bias [24]. We evaluated each study across the following domains:

**Sample representativeness:** Were isolates representative of the target population?
**Sample size adequacy:** Was the sample size sufficient for reliable molecular characterization?
**Sampling methods:** Were appropriate sampling methods used?
**Molecular methods:** Were validated, standardized molecular methods used?
**Quality control:** Were adequate quality control measures applied?
**Data analysis:** Were appropriate bioinformatics and statistical methods used?
**Reporting:** Were the results reported with sufficient detail?

Each domain was evaluated as low, moderate, or high risk of bias. Studies with high risk of bias in three or more domains were considered to be at overall high risk of bias.

### 2.7. Effect Measures and Synthesis Methods

We planned to perform a narrative synthesis as well as a meta-analysis. We organized findings according to:

**Prevalence of *Salmonella*** across the ME countries.
**Country and source-wise** distribution of specific ARGs.
**Resistance gene prevalence**: Collective prevalence estimates with 95% confidence intervals were calculated for common ARGs, grouped by source (food production vs. clinical) and country.
**Genetic similarity indicators**: Synthesized evidence on shared STs.
**Mobile genetic elements**: Summarized data on plasmids, integrons, and transposons carrying ARGs, with attention to elements found in both sources.
**Geographic patterns**: Examined regional variation in resistance gene distributions and gene types.
**Prevalence of MDR** across ME countries.

In addition, we assessed variability qualitatively by examining differences in study populations, molecular methods, and geographical settings. In addition, proportional meta-analysis was performed using OpenMeta [Analyst, version 1.0] [25], which is a free, open-source software that uses a random effects model (DerSimonian-Laird), applies a transformation to prevalence proportions, and uses a continuity correction approach for studies with zero-event cells (where applicable). For this purpose, we used events and the total sample size for each variable (country-wise, source-wise [food production environment and clinical isolates], and variant-wise). Heterogeneity was assessed using I-statistics and rated as very low (0–24%), low (25–49%), moderate (50–74%), and high (>74%) [26].

### 2.8. Certainty of Evidence

We assessed the confidence of data for main outcomes using the Grading of Recommendations Assessment Development and Evaluation (GRADE) framework adapted for prevalence and molecular epidemiology studies. We assessed confidence using five categories [27]:

**Risk of bias**: Reduced if most studies have moderate or high risk of bias.
**Variation**: Reduced if there is significant variability in results across studies.
**Indirect:** Reduced if the evidence was indirect.
**Inaccuracy**: Reduced if the sample size was small.
**Publication bias**: Assessed through funnel plots (when ≥10 studies) and consideration of selective reporting.

Evidence was categorized as high (⊕⊕⊕⊕), moderate (⊕⊕⊕○), low (⊕⊕○○), or very low (⊕○○○) certainty.

## 3. Results

### 3.1. Literature Search

A three-stage PRISMA flowchart was used for the selection of relevant studies. In the first stage, 1135 studies were identified and retrieved from different electronic databases, and 172 duplicate studies were excluded. In the second phase, 963 studies were screened by title and abstract, 843 irrelevant studies were excluded, and the remaining 120 studies were processed for full-text screening. Each study was evaluated according to the eligibility criteria, and 26 studies did not fulfill the criteria, with the reasons explained in the PRISMA flowchart (Figure 1). In the third stage, 94 selected studies were included for further narrative and quantitative synthesis.

### 3.2. General Characteristics of Included Studies

Table 1 summarizes the characteristics of the studies included in the review, demonstrating that most studies followed a cross-sectional study design due to the nature of the studies (surveys) conducted across ME countries, particularly Egypt (47/94, 50%), Iran (21/94, 22.34%), Saudi Arabia (7/94, 7.44%), Iraq (5/94, 5.31%), the UAE (5/94, 5.31%), Jordan (3/94, 3.19%), Lebanon (2/94, 2.12%), Kuwait (1/94, 1.06%), Palestine (1/94, 1.06%), Qatar (1/94, 1.06%), and one study performed both in Kuwait and the UAE [1/94, 1.06%] (Table 1). Most studies in the food production environment focused on animal sources (61/94, 64.89%), followed by both animal and human sources (15/94, 15.95%), and 2 (2.12%) studies isolated *Salmonella* serovars from animal, human, and environmental sources. Human sources (12/94, 12.76%) [28,29,30,31,32,33,34,35,36,37,38,39] were selected for clinical isolates. Among animal sources, poultry broilers were the most commonly visited source, although several also investigated dairy products [40,41,42,43,44,45,46], fish [47], environmental samples [48], or combinations of these sources [49,50].

Sample sizes varied considerably, ranging from 2 samples [51] to >3000 samples [52] in food production environments and clinical isolates, reflecting substantial diversity in study scale. The most frequently reported *Salmonella* serovars were *Enteritidis*, *Typhimurium*, *Infantis*, and *Kentucky*, while other serovars, such as *Minnesota*, *Heidelberg*, *Newport*, *Derby*, *Virchow*, and *Muenchen* were reported less commonly in food production environments and clinical isolates (Table 1). In the food production environments, samples were collected from a wide range of sources, including meat, eggs, carcasses, cloacal and fecal swabs, internal organs, dairy products, human handlers’ hand swabs, stool, environmental water, soil, vegetables, and food-contact surfaces, as described in Table 1. Samples from clinical environments were collected from stools, urine, and blood (Table 1). Most studies in food production environments did not specify the infection status of the sampled population. However, clinical isolates were obtained from patients with infections, such as diarrhea, gastroenteritis, and foodborne illness (Table 1). Overall, the included studies highlight the broad geographic distribution of studies across the ME region, diverse sampling strategies, and a wide range of host and environmental reservoirs investigated for *Salmonella* serovars across the ME region, with poultry-related sources representing the predominant focus of research.

**Table 1:** Summary of the general characteristics of the included studies.

Study ID	Country	Study Design	Sample Size	Type	Reported Serovars	Infection Type	Sampling Method
Food production environment							
Animal							
[53]	Egypt	Cross-sectional	220	Poultry broilers	*Typhimurium*, *Enteritidis*, *Infantis*	Diseased	Cloacal swabs and internal organs
[40]	Egypt	Cross-sectional	1600	Poultry broiler and dairy	*Typhimurium*, *Enteritidis*	NA	Meat and dairy products
[54]	Egypt	Cross-sectional	165	Poultry broiler	*Kentucky*, *Enteritidis*	NA	Skin, carcasses, raw egg yolks, eggshells, chicken feces
[55]	Egypt	Cross-sectional	200	Poultry broiler	*Enteritidis*, *Typhimurium*, *Kentucky*	NA	Carcasses, drumsticks, livers and gizzards
[56]	Iran	Cross-sectional	600	Poultry broiler	*Typhimurium*, *Landan*, *Saintpaul*, *Agona*, *Enteritidis*, *Kentucky*, *Newport*	NA	Carcasses
[57]	Egypt	Cross-sectional	300	Poultry broiler	*Typhimurium*	NA	Liver, heart, and spleen
[58]	Egypt	Cross-sectional	125	Poultry broiler	*Enteritidis*, *Typhimurium*, *Emek*, *Agona*	NA	Tables, knives, rinsing water, carcasses, and surfaces
[59]	Egypt	Cross-sectional	600	Poultry broiler	*Typhimurium*	NA	Breast meat and tool
[60]	Egypt	Cross-sectional	100	Poultry broiler	*Enteritidis*, *Typhimurium*, *Kentucky*	Diarrhea	Cloacal swab, liver, gallbladder, spleen, and intestinal content
[41]	Iran	Cross-sectional	34	Poultry and dairy	*Enteritidis*	NA	Meat
[61]	Egypt	Cross-sectional	NA	Poultry broiler	*Kentucky*, *Enteritidis*, *Typhimurium*	NA	NA
[42]	Egypt	Cross-sectional	180	Poultry and dairy	*Typhimurium*, *Paratyphi*, *Enteritidis*, *Kentucky*	NA	Meat and internal organs
[62]	Egypt	Cross-sectional	615	Poultry broiler	*Enteritidis*, *Typhimurium*	NA	Liver, intestinal content and gallbladder
[63]	Iran	In vitro	46	Poultry broiler	*Infantis*	NA	Previously isolated strains
[64]	Iraq	Cross-sectional	400	Poultry broiler	*Typhimurium*, *Enteritidis*, *Kentucky*, *Newport*, *Muenchen*	NA	Packaged frozen poultry carcasses
[43]	Egypt	Cross-sectional	150	Dairy meat	*Enteritidis*	NA	Fresh minced meat, smoked sausage and fresh beef burger
[65]	Egypt	Cross-sectional	353	Poultry broiler	*Enteritidis*, *Infantis*, *Kentucky*, *Maloma*, *Bardo*, *Gdansk*, *Typhimurium*, *Blegdame*	NA	Liver, yolk, gallbladder and caecum
[66]	Egypt	Cross-sectional	420	Poultry broiler	*Enteritidis*, *Typhimurium*, *Kentucky*, *Molade*	NA	Cloacal swabs, farm surrounding samples and freshly dressed chicken carcasses
[67]	Egypt	Cross-sectional	222	Chicken, pigeons, quails and ducks	*Enteritidis*, *Typhimurium*	NA	Internal organs
[68]	Egypt	Cross-sectional	120	Poultry broiler	*Enteritidis*, *Typhimurium*	NA	NA
[69]	Lebanon	Cross-sectional	133	Poultry broiler	*Kentucky*	NA	NA
[70]	Egypt	Cross-sectional	Healthy = 175, diseased chicken = 126	Poultry broiler	*Typhimurium*, *Kentucky*, *Infantis*	NA	Cloacal swab
[52]	Iran	Cross-sectional	3125	Poultry broiler	*Enteritidis*	NA	Meat and eggs
[47]	Egypt	Cross-sectional	200	Fish	*Kentucky*, *Enteritidis*, *Typhimurium*	NA	Meat
[71]	Egypt	Cross-sectional	391	Duckling, chick, turkey	*Enteritidis*, *Typhimurium*	NA	Imported meat
[72]	Egypt	Cross-sectional	300	Duck, pigeon, and quail carcasses	*Typhimurium*, *Kentucky*, *Enteritidis*	NA	NA
[73]	Egypt	Cross-sectional	500	Poultry broiler	*Salmonella serovars*	NA	Liver, yolk sac, cecum, spleen and heart
[44]	Egypt	Cross-sectional	400	Poultry and dairy	*Infantis*, *Enteritidis*, *Typhimurium*	NA	Meat and carcasses swabs
[74]	Iraq	Cross-sectional	2000	Poultry broiler	*Enteritidis*	NA	Meat and eggs
[75]	Iraq	Cross-sectional	185	Poultry broiler	*Enteritidis*	NA	Fecal cloacal swabs
[76]	Iraq	Cross-sectional	250	Poultry broiler	*Enteritidis*	NA	Imported and local raw chicken meat and liver
[51]	Saudi Arabia	Cross-sectional	2	Poultry broiler	*Minnesota*	NA	Meat
[77]	Iran	Cross-sectional	150	Poultry broiler	*Salmonella serovars*	NA	Meat
[78]	Egypt	Cross-sectional	832	Poultry broiler	*Enteritidis*, *Shangani*, *Gueuletapee*	NA	Liver, intestinal content, spleen, and gallbladder
[79]	Egypt	Cross-sectional	110	Animal	*Salmonella serovars*	NA	Milk, cheese, yogurt, ice cream
[45]	Egypt	Cross-sectional	200	Poultry and dairy	*Typhimurium*, *Enteritidis*, *Kentucky*	NA	Fecal and rectal swabs from diarrhea calves, dead chickens
[80]	Iran	Cross-sectional	100	Poultry broiler	*Salmonella serovars*	NA	Cloacal swab sample
[81]	Saudi Arabia	Cross-sectional	39	Poultry broiler	*Minnesota*, *Infantis*, *Enteritidis*, *Kentucky*	NA	Meat
[82]	Egypt	Cross-sectional	150	Poultry broilers	*Enteritidis*, *Typhimurium*	Diseased	Meat
[83]	Egypt	Cross-sectional	246	Vaccinated poultry broiler	*Enteritidis*	NA	Cecum, liver, gallbladder, air sacs, spleen, and pericardium
[84]	UAE	Cross-sectional	254	Poultry broiler	*Minnesota*, *Heidelberg*, *Kentucky*	NA	Imported meat
[85]	UAE	Cross-sectional	315	Poultry broiler	*Infantis*, *Minnesota*, *Kentucky*, *Brancaster*, *Livingstone*	NA	Frozen meat
[86]	Jordan	Cross-sectional	635	Poultry broiler	*Infantis*, *Enteritidis*, *Typhimurium*, *Choleraesuis*, *Hadar*, *Dublin*, *Gallinarum*	NA	Feed mill, drag swabs, paper lines, cloacal swabs, neck skins
[87]	Iran	Cross-sectional	NA	Poultry broiler	*Typhimurium*, *Enteritidis*, *Infantis*	NA	Fecal samples
[88]	Egypt	Cross-sectional	150	Poultry broiler	*Salmonella serovars*	NA	Meat
[89]	Egypt	Cross-sectional	800	Poultry broiler	*Grampian*, *Kentucky*, *Blegdam*	NA	Heart, liver, intestine, and yolk sac
[90]	Egypt	Cross-sectional	129	Poultry broiler	*Salmonella serovars*	NA	Yolk sac contents
[91]	Iran	Cross-sectional	132	Poultry broiler	*Infantis*, *Typhimurium*, *Enteritidis*	NA	Carcasses
[92]	Jordan	Cross-sectional	200	Poultry broiler	*Salmonella serovars*	NA	Meat
[93]	Iran	Cross-sectional	500	Poultry broiler	*Infantis*, *Enteritidis*, *Typhimurium*	NA	Eggs
[94]	Jordan	Cross-sectional	700	Poultry broiler	*Typhimurium*, *Enteritidis*	NA	Meat
[95]	UAE	Cross-sectional	50	Poultry broiler	*Infantis*	NA	Caecal droppings
[96]	Iran	Cross-sectional	1274	Poultry broiler	*Enteritidis*	NA	Swabbing
[46]	Iran	Cross-sectional	680	Poultry broiler and dairy	*Typhimurium*, *Enteritidis*	NA	Meat, chopping board, swabs, knife swabs, dressing water
[97]	Egypt	Cross-sectional	450	Poultry broiler	*Kentucky*, *Derby*, *Typhimurium*	NA	Healthy, diseased, and dead carcasses
[98]	Saudi Arabia	Cross-sectional	260	Poultry broiler	*Enteritidis*, *Heidelberg*, *Bareilly*, *Cerro*, *Typhimurium*	NA	Eggs
[99]	UAE	Cross-sectional	380	Poultry broiler	*Enteritidis*, *Indiana*, *Minnesota*	NA	Eggs
[100]	Saudi Arabia	Cross-sectional	859	Poultry products	*Minnesota*	NA	Processed and unprocessed meat
[101]	Egypt	Cross-sectional	30	Poultry broiler	*Enteritidis*, *Give*	NA	Meat
[102]	Egypt	Cross-sectional	240	Poultry broiler	*Salamae*, *Enteritidis*, *Infantis*	NA	Meat
[103]	Egypt	Cross-sectional	200	Poultry broiler	*Kentucky*, *Typhimurium*, *Salamae*, *Infantis*	NA	Cloacal swabs, liver, spleen, heart, unabsorbed yolk sacs, and intestinal contents
Animal and human							
[104]	Egypt	Cross-sectional	150	Human, poultry broiler and duck	*Typhimurium*, *Derby*, *Kiel*, *Rubislaw*	Diarrhea	Meat and patients’ stool
[105]	Iran	Cross-sectional	26	Poultry, dairy and human	*Enteritidis*	Diarrhea	Meat and human stool and blood
[106]	Egypt	Cross-sectional	115	Dairy and handlers	*Salmonella serovars*	Diarrhea	Stool from diarrheic calves and hand swabs from calf handlers
[107]	Iran	Cross-sectional	Chicken meat = 100, Stool samples = 400	Poultry and patients	*Salmonella serovars*	Community-acquired diarrhea	Meat and patients’ stool
[108]	Egypt	Cross-sectional	Chicken: 200, Human: 25	Poultry and humans	*Enteritidis*, *Kentucky*	NA	Meat and human stool
[109]	Egypt	Cross-sectional	Chicken = 1210, Humans = 375	Chicken and humans	*Typhimurium*	Diarrhea	Fecal dropping and cloacal swabs, liver, spleen, cecum, gallbladder, and yolk sac from dead birds. From humans, stool was used
[110]	Iran	Cross-sectional	595	Poultry, dairy products and human	*Infantis*, *Enteritidis*	NA	Meat and human stool
[111]	Egypt	Cross-sectional	Chicken = 300, Human samples = 60	Poultry broiler and human	*Enteritidis*, *Typhimurium*	NA	Meat and human stool
[112]	Egypt	Cross-sectional	Chicken meal = 150, Food handlers = 100, Patients = 50	Poultry broiler and humans	*Typhimurium*, *Enteritidis*, *Kentucky*	Diarrhea	Chicken ready-meal and human hand swabs
[113]	Egypt	Cross-sectional	Chicken = 180, Humans = 60	Poultry broiler and humans	*Typhimurium*, *Enteritidis*, *Kentucky*	NA	Liver, internal organs, and human hand swab
[114]	Egypt	Cross-sectional	129	Poultry broiler and humans	*Bargny*, *Kentucky*, *Virchow*, *Enteritidis*, *Typhimurium*, *Infantis*, *Abo*, *Irumu*	NA	Chicken giblets, water tanks, and workers’ hand swabs
[115]	Palestine	Cross-sectional	592	Poultry broiler and human	*Muenchen*	Gastroenteritis	Meat and human stool
[116]	Egypt	Cross-sectional	800	Animal-based products and human	*Typhimurium*, *Enteritidis*	NA	Dairy products and human hand swabs
[117]	Egypt	Cross-sectional	150	Dairy and human	*Typhimurium*, *Infantis*, *Kentucky*	NA	Rectal swabs, meat swabs, and hand swabs
[118]	Egypt	Cross-sectional	13	Broiler chickens and humans	*Typhimurium*, *Enteritidis*, *Infantis*, *Virchow*, *Kentucky* *Peniscola*, *Grampian*, *Gueuletapee*	Diarrhea, gastroenteritis	Gallbladder, liver, bone marrow, intestine, and human stool
Human and environment							
[119]	Saudi Arabia	Cross-sectional	100	Human and environment	*Enteritidis*, *Typhimurium*, *Arizona*	NA	Stool and agricultural products
[120]	Lebanon	Cross-sectional	NA	Human and foods	*Enteritidis*, *Typhimurium*	Foodborne illness	Stool and agricultural products
[121]	Egypt	Cross-sectional	501	Food and human	*Enteritidis*	NA	Raw food, ready-to-eat food/drink, human blood, stool and hand swabs
Environment							
[48]	UAE	Cross-sectional	400	Vegetables	*Salmonella serovars*	NA	Leaves and edible parts
Human, animal and environment							
[49]	Iraq	Cross-sectional	365	Human, animal and environment	*Salmonella serovars*	Diarrhea	Stool, rectal swabs of infected chickens with diarrhea, soil, and water bodies
[50]	Saudi Arabia	Cross-sectional	770	Poultry, humans and environment	*Typhimurium*, *enteritidis*, *Infantis*	NA	Stool from poultry birds and internal organs, human stool, vegetables and water sources
Clinical isolates							
Humans							
[29]	Iran	Cross-sectional	2116	Humans	*Enteritidis*	Gastroenteritis	Stool
[31]	Kuwait and UAE	Cross-sectional	407	Humans	*Typhimurium*, *Enteritidis*	Acute diarrhea	Stool
[30]	Egypt	Cross-sectional	NA	Humans	*Grampian*, *Larose*, *Hato*, *Texas*	Diarrhea	Stool
[32]	Iran	Cross-sectional	202	Humans	*Typhimurium*, *Bredeney*, *Infantis*	Gastroenteritis	Stool
[28]	Iran	Cross-sectional	2700	Humans	*Enteritidis*	Gastroenteritis	Stool
[39]	Iran	Cross-sectional	2200	Humans	*A*, *B*, *C*, *D*	Diarrhea	Stool
[35]	Saudi Arabia	Cross-sectional	200	Humans	*Enteritidis*, *Typhimurium Livingstone*, *Kentucky*, *Poona*	Gastroenteritis	Stool, urine, tissue, abdominal fluid, wound, and blood
[33]	Iran	Cross-sectional	230	Humans	*Salmonella serovars*	Diarrhea	Stool
[38]	Iran	Cross-sectional	NA	Humans	*Infantis*, *Enteritidis*	NA	Stools, blood, wounds, urine, or other biological fluids
[37]	Qatar	Cross-sectional	NA	Humans	*Typhi*, *Typhimurium*, *Enteritidis*	Diarrhea	Blood
[34]	Kuwait	Cross-sectional	167	Healthy and patients	*Enteritidis*, *Typhimurium*, *Kentucky*, *Newport*	Diarrhea	Stool and extra-intestinal samples
[36]	Iran	Cross-sectional	NA	Human	*Enteritidis*	NA	Stool, and blood urine

Abbreviations: NA = not applicable.

### 3.3. Characteristics of Molecular Methods and Resistant Genes

Different molecular approaches were used in food production environments and clinical isolates to characterize ARGs in *Salmonella* isolates, and revealed that PCR-based methods, such as uniplex PCR, multiplex PCR, RT PCR, ERIC-PCR, and PFGE, were overwhelmingly the most frequently employed techniques, while a limited number of studies incorporated WGS in the case of food production environments [48,51,64,69,81,84,85,95,98,99,100,115]. Only two studies used WGS for clinical isolates [34,37]. Additionally, few studies also used ST (MLST) for further characterization of food production environment and clinical isolate’s AMR genes [34,51,69,81,115]. The findings demonstrate a high diversity of classes of resistance, with β-lactamase genes being the most commonly detected, particularly *bla_TEM_*, *bla_CTXM_*, *bla_SHV_*, *bla_CMY_*, *bla_OXA_*, and *bla_NDM_*, and extended-spectrum β-lactam (ESBL) antibiotics in both types of isolates (Table 2). Resistance determinants against other important antimicrobial classes were also frequently reported, including quinolone resistance genes (*qnrA*, *qnrB*, *qnrS*, *gyrA*, *parC*), tetracycline resistance genes (*tetA*, *tetB*, *tetC*, *tetG*, *tetK*), sulfonamide and trimethoprim resistance genes (*sul1*, *sul2*, *sul3*, *dfrA* variants), and aminoglycoside resistance genes (*aadA*, *aac*, *aph*, *ant* families), as described in Table 2. Several studies additionally identified resistance genes associated with chloramphenicol/florfenicol (*floR*, *cat1*), colistin (*mcr-1*, *pmrA*, *pmrB*), macrolides (*ereA*, *ermB*, *mphA/B*), and carbapenems (*blaKPC*, *blaNDM*) in both tested isolates, highlighting the emergence of multidrug-resistant (MDR) and clinically significant strains. Studies utilizing WGS generally detected a broader spectrum of resistance determinants than conventional PCR-based investigations, emphasizing the growing value of genomic surveillance for comprehensive antimicrobial resistance monitoring.

### 3.4. Genotyping Outcomes

In Table 3, we summarize the genotyping characteristics of *Salmonella* isolates and highlight the important role of plasmids, integrons, transposons, and STs in the dissemination of AMR across the included studies. Most studies in food production environments reported resistance determinants to be located predominantly on plasmids, although several studies identified genes on both plasmid and chromosomal DNA [40,57,61,69,100,104,108], suggesting the involvement of both horizontal and vertical transmission mechanisms. Among the mobile genetic elements, class 1 integrons were frequently detected [45,59,60,67,103,104], followed by class 2 and class 3 integrons, while a smaller number of studies reported the presence of specific plasmid types, emphasizing their contribution to the acquisition and spread of resistance genes in food production environment isolates (Table 3). ST data revealed considerable genetic diversity among isolates, with recurrent identification of internationally recognized lineages, such as ST11, ST15, ST19, ST32, ST198, ST548, ST58, and ST22, alongside several novel or less common STs, as described in Table 3. Notably, ST198, a lineage often associated with MDR, was reported across multiple studies, while ST11, ST15, ST19, and ST32 were also repeatedly identified in different geographical settings (Table 3). In clinical isolates, resistance determinants are located predominantly on plasmids [32,33,38], while one study found them on both chromosomal and plasmids [35]. Additionally, ST data revealed ST1, ST10, ST58, ST183, ST1918, ST19, ST29, ST198, ST158, and ST166 in clinical isolates (Table 3). Meanwhile, detailed characterization of mobile genetic elements was limited in many studies. Few studies provided complete plasmid sequence analysis and applied conjugation experiments to confirm the transfer of resistance plasmids in food production environments and clinical isolates. Meanwhile, direct comparative genomic evidence based on contemporaneous, geographically linked isolates from different sources was also largely unavailable. Only STs (ST19 and ST198) were found in both food production environment and clinical isolates.

### 3.5. Phenotypical Antibiotic Resistance Status

We summarize the phenotypic AMR patterns of *Salmonella* isolates reported across GCC countries and neighboring ME regions in isolates collected from food production environments and clinical settings, revealing a widespread and concerning burden of MDR. In food production environments, isolates with the highest resistance rates were consistently observed against ampicillin, amoxicillin/amoxicillin-clavulanic acid, tetracyclines, nalidixic acid, trimethoprim-sulfamethoxazole, streptomycin, and chloramphenicol, with several studies reporting resistance frequencies exceeding 80–100% for these agents [40,47,53,62]. Resistance to fluoroquinolones, including ciprofloxacin and norfloxacin, was also frequently documented, although prevalence varied significantly between studies, indicating the ongoing emergence of reduced susceptibility to critically important antimicrobials used for severe salmonellosis [61,105]. Furthermore, several studies reported resistance to third-generation cephalosporins such as cefotaxime, ceftazidime, and ceftriaxone, suggesting the circulation of ESBL-producing serovars [42,70,97,111]. Alarmingly, resistance to last-resort antibiotics, including colistin, imipenem, and meropenem, was also identified in some studies, although at lower frequencies, indicating the emergence of highly resistant lineages [74,101]. In clinical isolates, high resistance was observed against ampicillin [30,31,34,36]. Meanwhile, other antibiotics also demonstrated varying degrees of resistance, as described in Table 4. Across food production environments and clinical isolates, MDR profiles were common, with many serovars exhibiting simultaneous resistance to multiple antimicrobial classes, including β-lactams, tetracyclines, aminoglycosides, sulfonamides, phenicol, and quinolones.

### 3.6. Meta-Analysis

#### 3.6.1. Country and Source-Wise Distribution of *Salmonella* Serovars

The highest prevalence estimates were reported in Kuwait (0.64) from one study, Saudi Arabia (0.45), and Jordan (0.40), whereas lower prevalence was observed in Lebanon (0.06), Palestine (0.03), Iraq (0.10), and the UAE (0.14). Meanwhile, Egypt and Iran, which contributed the largest number of studies, demonstrated pooled prevalences of 0.29 (95% CI, 0.21 to 0.37) and 0.35 (95% CI, 0.25 to 0.46), respectively, both of which were statistically significant (*p* < 0.001), as described in Table 5.

In the food production environment, the most frequent *Salmonella* serovars were detected in animal-based samples (0.31), followed by both animal and human sources (0.27). In contrast, lower prevalence was observed in vegetable samples (0.01), while studies incorporating environmental sources reported intermediate prevalence estimates ranging from 12% to 33% (Table 5). Clinical isolates showed a prevalence of 0.28, suggesting that food production environments remain the principal reservoir for *Salmonella* transmission in the region. The overall pooled prevalence of *Salmonella* was 0.30 (95% CI: 0.23–0.36), although heterogeneity was extremely high (I^2^ = 99.92%), indicating considerable differences among the included studies.

#### 3.6.2. Classes of Resistance, Country-Wise Distribution in ME Region

**Figure 2:**
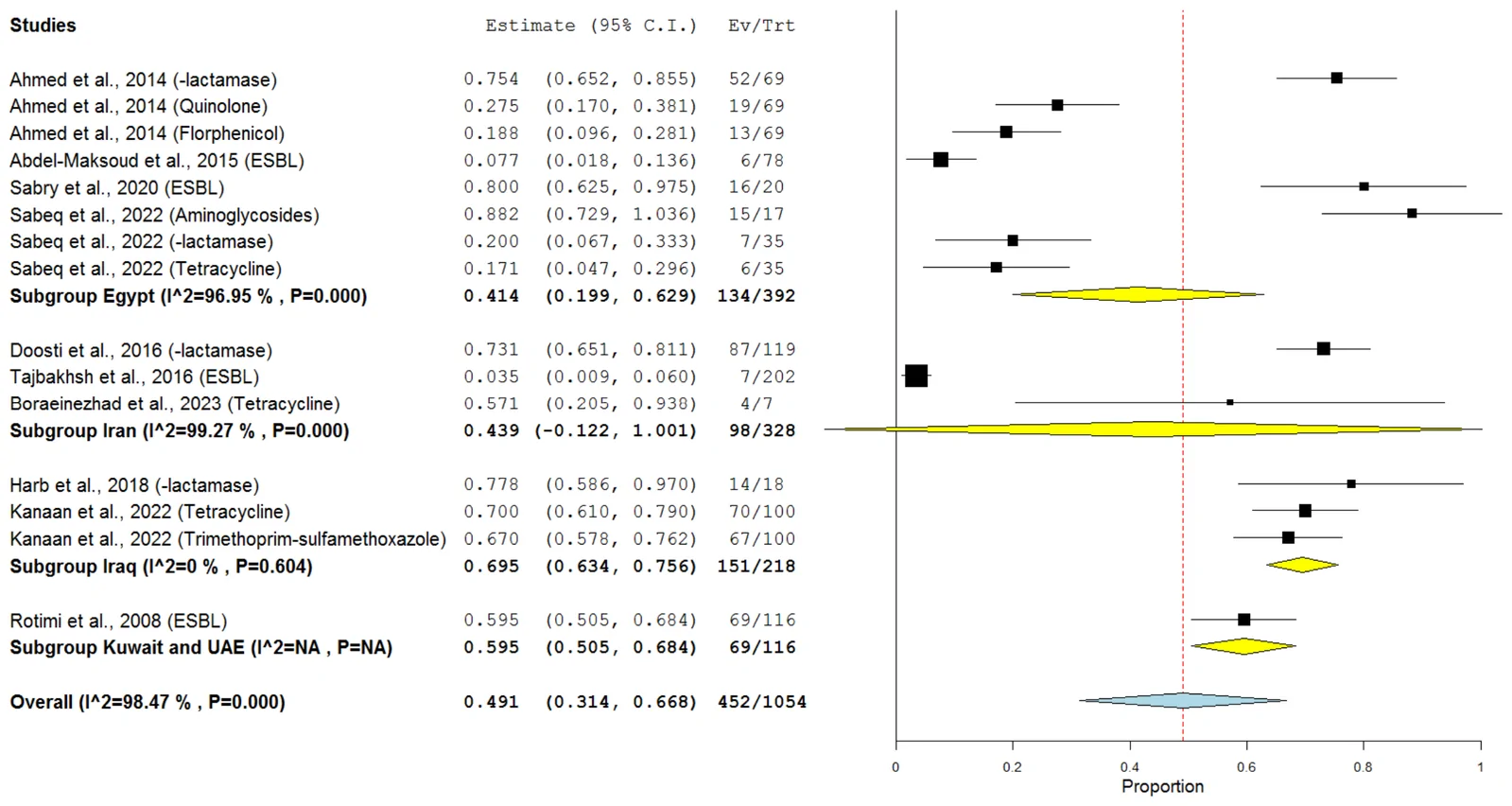
Forest plot showing the country-wise distribution of classes of resistance across the ME region [31,32,40,54,56,64,70,74,80,121].

The pooled prevalence of specific classes of resistance, such as β-lactamases including ESBLs, quinolones, florfenicol, aminoglycosides, and tetracyclines in Egypt was 0.41 (95% CI, 0.19 to 0.62, *p* < 0.001, I^2^ = 96.95%). In Iran, the pooled prevalence of β-lactamases, ESBLs, and tetracyclines was 0.43 (95% CI, −0.12 to 1, *p* = 0.12, I^2^ = 99.27%). In Iraq, a high prevalence of β-lactamase, tetracyclines, and trimethoprim-sulfamethoxazole was observed (0.69 [95% CI, 0.63 to 0.75]), with a significant difference (*p* < 0.001) and low heterogeneity (I^2^ = 0%). Meanwhile, as only a single study was conducted in Kuwait and the UAE, no analysis was performed. Overall, the prevalence of these specific classes was 0.49 (95% CI, 0.31 to 0.66), with a significant difference (*p* < 0.001); however, high heterogeneity (I^2^ = 98.47%) was observed among the included studies (Figure 2).

#### 3.6.3. Classes of Resistance, Source-Wise Distribution in ME Region

**Figure 3:**
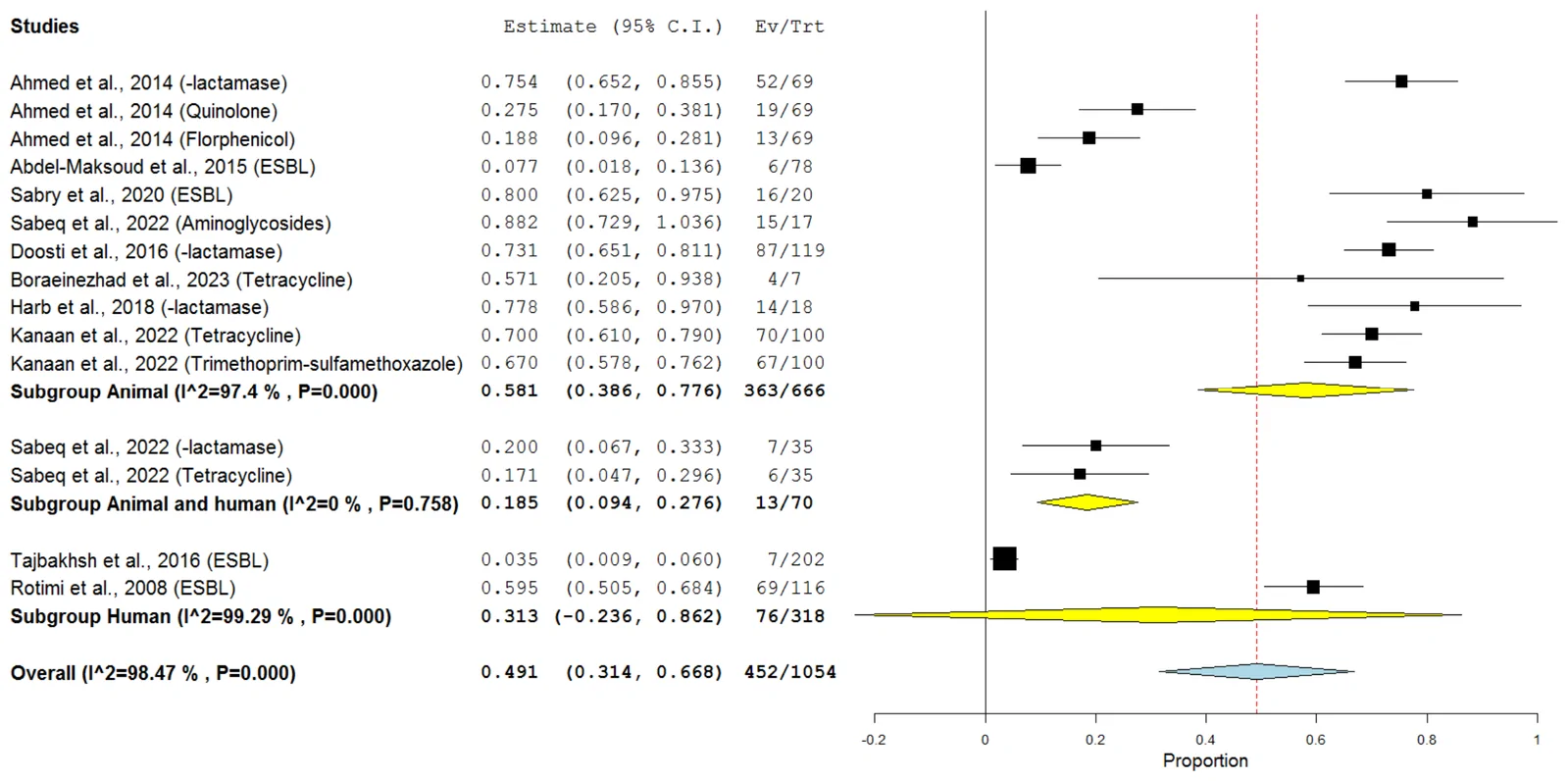
Forest plot showing the source-wise distribution of resistance classes across the ME region [31,32,40,54,56,64,70,74,80,121].

Most specific ARGs, such as β-lactamase including ESBLs, quinolone, florfenicol, tetracyclines, and trimethoprim-sulfamethoxazole, were isolated from food production environments, such as animal sources, with a pooled prevalence of 0.58 (95% CI, 0.38 to 0.77, *p* < 0.001); however, high heterogeneity (97.4%) was observed among the studies (Figure 3). Two studies reported a pooled prevalence of 0.18 for β-lactamase and tetracyclines in both animals and humans (*p* < 0.001, I^2^ = 0%). The pooled prevalence of ESBLs in clinical isolates (humans) was 0.31 (95% CI, −0.23 to 0.86), with a non-significant difference (*p* = 0.26) and high heterogeneity (I^2^ = 99.29%). Overall, the prevalence of these specific classes of resistance was 0.49 (95% CI, 0.31 to 0.66, *p* < 0.001, I^2^ = 98.47%), as described in Figure 3.

#### 3.6.4. Genotypic Resistance Pattern

##### β-Lactamase

A meta-analysis was conducted on the proportion (prevalence) estimates of *Salmonella* serovars with β-lactamase resistant genes across multiple studies, stratified by country-level subgroups (notably Egypt, Iran, Jordan, Saudi Arabia, UAE, Kuwait, and Qatar). The pooled prevalence for Egypt was 0.55 (95% CI 0.39 to 0.71). However, very high heterogeneity (I^2^ = 99.3%, *p* < 0.001) indicates substantial variability across studies. A high prevalence of β-lactamase resistant genes was observed in Iraq (0.69 [95% CI, 0.17 to 1.22]), followed by UAE (0.59 [95% CI, 0.42 to 0.76]), Saudi Arabia (0.56 [95% CI, 0.02 to 1.10), while the lowest prevalence was observed in Iran (0.35 [95% CI, 0.17 to 0.52]). Meanwhile, most subgroups had high heterogeneity (Supplementary Table S3).

In terms of source, a high prevalence of β-lactamase resistant genes was observed in food production environment isolates, such as isolates from animals (0.50 [95% CI, 0.37 to 0.63]), with a significant difference (*p* < 0.001); however, high heterogeneity (I^2^ = 99.34%) was observed among the included studies (Supplementary Table S3). The lowest prevalence was observed in clinical isolates (0.36 [95% CI, 0.21 to 0.52]), with a significant difference (*p* < 0.001) and high heterogeneity (98.59%), as described in (Supplementary Table S3).

Variant-wise distribution indicated that *bla_TEM_* had high prevalence (0.64 [95% CI, 0.52 to 0.77]), with a significant difference and high heterogeneity (I^2^ = 98.51%), followed by *bla_CMY_* (0.47 [95% CI, −0.05 to 1.00], I^2^ = 99.50%), with a non-significant difference (*p* = 0.07), as described in Supplementary Table S3. Overall β-lactamase variant prevalence in ME countries was 0.48 (95% CI, 0.37 to 0.58), with a significant difference. However, high heterogeneity (I^2^ = 99.34%) was also observed. This high heterogeneity is not explained by chance alone, supporting the use of a random-effects model and implying that differences in study populations, settings, or methodologies likely contribute to the observed dispersion of results.

##### Tetracycline

A high prevalence of tetracycline-resistant genes was found in Egypt (0.75 [95% CI, 0.60 to 0.90]), with a significant difference (*p* < 0.001) and high heterogeneity (I^2^ = 97.33%), followed by Iraq (0.63 [95% CI, 0.14 to 1.13], *p* = 0.01, I^2^ = 91.22%). The lowest prevalence was observed in the UAE (0.32 [95% CI, −0.04 to 0.68]), with a non-significant difference (*p* = 0.08) and high heterogeneity (I^2^ = 97.73%), as described in (Supplementary Table S3).

In the food production environment, a high prevalence was found in animal sources (0.51 [95% CI, 0.33 to 0.69]), with a significant difference (*p* < 0.001) and high heterogeneity (I^2^ = 99.67). Isolates collected from animals as well as humans working in that environment showed a prevalence of 0.47 (95% CI, 0.23 to 0.72, *p* < 0.001, I^2^ = 90.57%) (Supplementary Table S3).

In terms of variants, *tetA* had high prevalence (0.65 [95% CI, 0.52 to 0.79], *p* < 0.001, I^2^ = 98.27), followed by *tetC* (0.55 [95% CI, −0.23 to 1.36]), with a non-significant difference and high heterogeneity (I^2^ = 99.83). Overall, the prevalence of tetracycline variants in ME countries was 0.51 (95% CI, 0.34 to 0.67, *p* < 0.001, I^2^ = 99.62%), as described in (Supplementary Table S3).

##### Aminoglycoside

Across the ME countries, a high prevalence of aminoglycoside was observed in Egypt (0.57, 95% CI, 0.28 to 0.85, *p* < 0.001, I^2^ = 99.37%), followed by Iran (0.41, [0.09 to 0.72, *p* = 0.01, I^2^ = 98.37%]), while the lowest was observed in Iraq (0.27, [95% CI, 0.08 to 0.45, *p* = 0.004]), with low heterogeneity (I^2^ = 0%). The prevalence was even higher in the limited studies from the UAE (0.96) and vegetables (0.91), although these estimates were based on a small number of studies (one study) and samples (Supplementary Table S3).

In food production environments, animal sources had a prevalence of 0.43 (95% CI, 0.26 to 0.60, *p* < 0.001, I^2^ = 98.67%), and samples collected from both animals and humans working in the environment had a prevalence of 0.55 (95% CI, 0.19 to 0.90, *p* = 0.002, I^2^ = 99.56%). One study had the required data for clinical isolates, which showed a prevalence of 0.96 (95% CI, 0.88 to 1.05) (Supplementary Table S3).

Among aminoglycoside variants, *aadA2* and *aac(6′)-Iaa* had a high prevalence (0.99, 95% CI, 0.97 to 1.01) and (0.96, 95% CI, 0.88 to 1.04), with a significant difference and low heterogeneity (I^2^ = 0%), as described in Supplementary Table S3. Overall, the prevalence of aminoglycosides in ME countries was 0.51 (95% CI, 0.33 to 0.69, *p* < 0.001, I^2^ = 99.43%) (Supplementary Table S3). This considerable variation in prevalence estimates was observed across individual studies, ranging from very low to extremely high values, reflecting differences in geographical locations, host species, sampling strategies, diagnostic methods, and study designs. The heterogeneity was substantial (I^2^ = 99.43%, *p* < 0.001), indicating marked variability among studies.

##### Quinolone

A high pooled prevalence of quinolone gene resistance was found in Iran (0.47; 95% CI, 0.07 to 0.87), with a significant difference (*p* = 0.02), but it demonstrated high heterogeneity (I^2^ = 97.66%), as described in Supplementary Table S3. Likewise, in Egypt, the pooled prevalence was 0.40 (95% CI, 0.18 to 0.62), with a significant difference (*p* < 0.001) and high heterogeneity (97.47%) (Supplementary Table S3).

In the food production environment, the pooled prevalence of quinolone resistant genes was 0.30 (95% CI, 0.18 to 0.43, *p* < 0.001, I^2^ = 94.34%) in isolates collected from animal sources. In contrast, isolates collected from clinical settings indicated a prevalence of 0.52 (95% CI, 0.18 to 0.87, *p* = 0.003, I^2^ = 97.17%) (Supplementary Table S3).

Overall, five variants of quinolone-resistant genes were found in ME countries, with a high pooled prevalence of 0.50 (95% CI, 0.24 to 0.76, *p* < 0.001, I^2^ = 96.6%) for *qnrS*, followed by *qnrA* (0.37, [95% CI, 0.00 to 0.75]), with a non-significant difference (*p* = 0.05) and high heterogeneity (98.31%). Overall, the pooled prevalence of quinolone resistant genes in ME countries was 0.40 (95% CI, 0.25 to 0.55, *p* < 0.001, I^2^ = 97.26%), as described in (Supplementary Table S3).

##### Sulfonamide

Qatar demonstrated a high pooled prevalence of sulfonamide-resistant genes (0.91, 95% CI, 0.80 to 1.01, *p* < 0.001), with low heterogeneity (I^2^ = 0%), followed by Egypt (0.72, 95% CI, 0.48 to 0.95, *p* < 0.001, I^2^ = 99.54%). The lowest prevalence was observed in the UAE (0.34, 95% CI, −0.09 to 0.78), with a non-significant difference (*p* = 0.12); however, it showed high heterogeneity (I^2^ = 98.55) (Supplementary Table S3).

In food production environments, isolates collected from both animals and humans showed a high pooled prevalence for sulfonamide resistant genes (0.73, 95% CI, 0.24 to 1.22, *p* = 0.003, I^2^ = 94.64%). Isolates from animal sources demonstrated a prevalence of 0.52 (95% CI, 0.31 to 0.74, *p* < 0.001, I^2^ = 99.53%). Isolates from clinical settings showed a prevalence of 0.88 (95% CI, 0.77 to 0.99, *p* < 0.001), with moderate heterogeneity (I^2^ = 30.58%) (Supplementary Table S3).

Sulfonamide-resistant gene variants showed that sul1 had a high prevalence in GCC countries (0.65, 95% CI, 0.46 to 0.84, *p* < 0.001, I^2^ = 99.17%), followed by sul3 (0.64, 95% CI, −0.01 to 1.30), with a non-significant difference (*p* = 0.05); however, high heterogeneity (I^2^ = 99.36) was also observed among the included studies. Overall, the pooled prevalence of sulfonamide in these countries was 0.60 (95% CI, 0.43 to 0.77, *p* < 0.001, I^2^ = 99.36%), as described in (Supplementary Table S3).

##### Chloramphenicol

Only three countries showed resistant genes associated with chloramphenicol, with high pooled prevalence in Iran (0.98, 95% CI, 0.96 to 1.01, *p* < 0.001, I^2^ = 0%), followed by Egypt (0.85, 95% CI, 0.69 to 1.01, *p* < 0.001, I^2^ = 92.02%), while Jordan had 0.35 (95% CI, −0.26 to 0.97), with a non-significant difference (*p* = 0.26) and high heterogeneity (I^2^ = 99.65%) (Supplementary Table S3).

In food production environments, a high pooled prevalence of chloramphenicol resistant genes was observed in isolates collected from both animals and humans working in the food production environment (0.78, 95% CI, 0.63 to 0.92, *p* < 0.001), with moderate heterogeneity (I^2^ = 46.02). Animal sources also indicated a high prevalence (0.74, 95% CI, 0.35 to 1.13, *p* < 0.001, I^2^ = 99.85%) (Supplementary Table S3).

Two variants of chloramphenicol resistance genes were found across ME countries. High pooled prevalences of floR (0.81, 95% CI, 0.65 to 0.97, *p* < 0.001, I^2^ = 94.97) and cat1 (0.67, 95% CI, 0.02 to 1.31, *p* < 0.04, I^2^ = 99.93) were observed. Overall prevalence was 0.76 (95% CI, 0.43 to 1.09, *p* < 0.001, I^2^ = 99.79%) (Supplementary Table S3).

#### 3.6.5. Phenotypical Resistance (MDR) Across ME Countries

**Figure 4:**
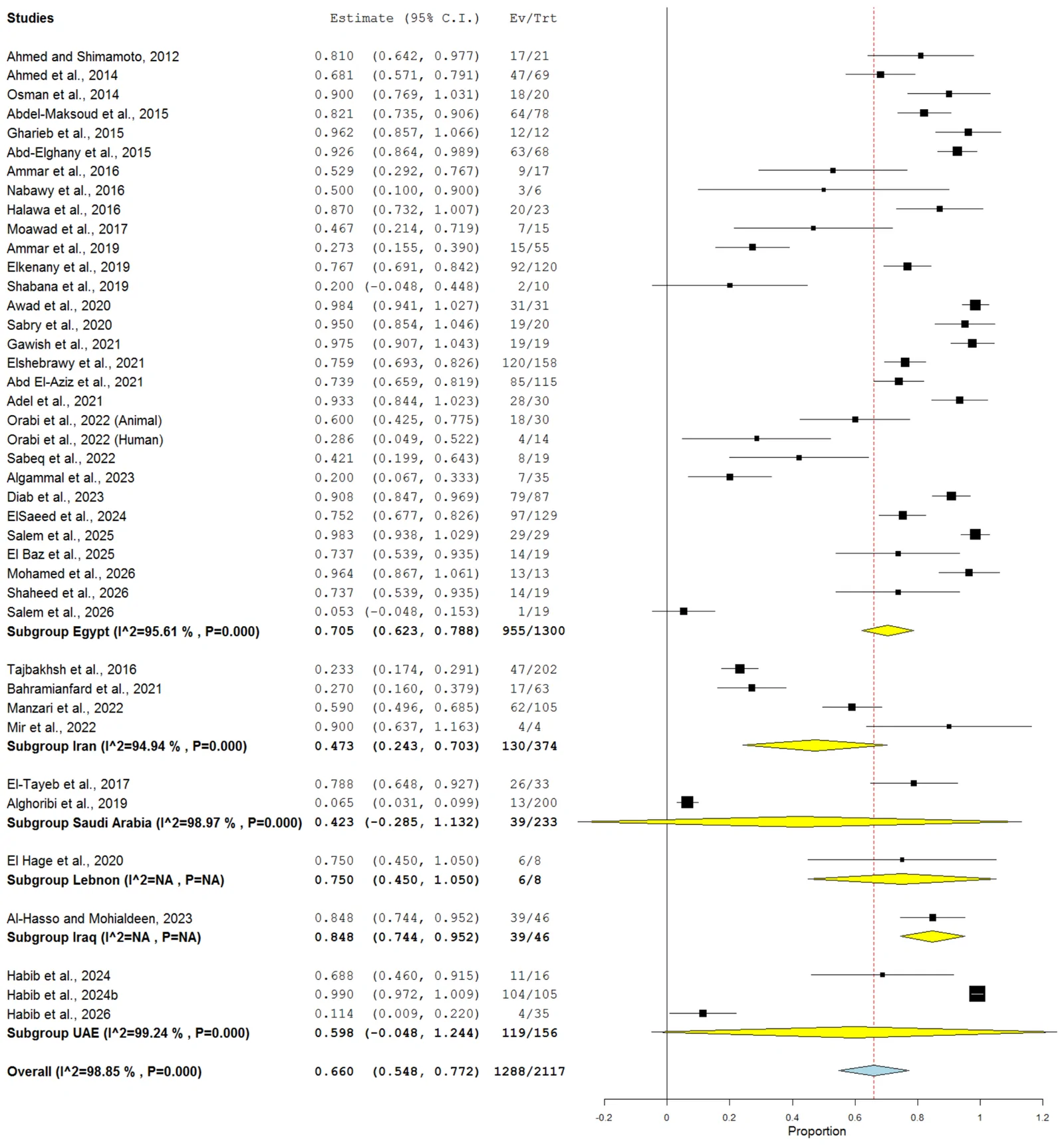
Forest plot for the prevalence of MDR in GCC and ME countries [30,32,35,40,42,44,47,49,52,53,54,55,57,58,60,65,66,67,69,70,72,77,82,84,85,88,97,99,102,103,104,108,109,110,111,116,117,118,119,121].

Overall, MDR was 0.66 (95% CI, 0.54 to 0.77, *p* < 0.001) in ME countries, particularly in Egypt (0.70, 95% CI, 0.62 to 0.78, *p* < 0.001), Iran (0.47, 95% CI, 0.24 to 0.70, *p* < 0.001), Saudi Arabia (0.42, 95% CI, −0.28 to 1.13, *p* = 0.24), and UAE (0.59, 95% CI, −0.04 to 1.24, *p* = 0.07). Meanwhile, high heterogeneity (I^2^ = 98.85%) was observed among the included studies (Figure 4).

### 3.7. Results of the Methodological Quality Assessment

Our risk of bias assessment demonstrates variable quality among the included studies. Overall, 62 studies (65.25%) had low risk of bias, 1 study (1.06%) had moderate risk of bias, and 32 studies (32.97%) had high risk of bias.

**Sample size, adequacy and methods:** Most studies (*n* = 92, 97.87%), (*n* = 90, 95.74%), and (*n* = 88, 93.61%) used a suitable sample size, sample adequacy, and sampling method, respectively. All these studies had a low risk of bias (Table 6).
**Molecular methods and quality control:** Most of the studies (*n* = 90, 95.74%) used validated molecular methods and were categorized as low risk in this domain. However, quality control reporting was inconsistent. Only 43 studies (45.74%) clearly described quality control measures for sequencing or molecular typing, while 51 studies (54.25%) provided no quality control data (Table 6).
**Data analysis and reporting**: Bioinformatics data were overall well described; however, an appropriate statistical analysis section was missing in most studies (*n* = 24, 25.53%, and *n* = 26, 27.65%), which had moderate and high risk of bias, respectively. Although all studies reported resistance gene prevalence, AMRs, and ARGs, only 2 studies (2.12%) demonstrated insufficient detail on genetic data.

Risk of bias assessments for individual studies are summarized in Supplementary Table S3, with detailed assessments provided in Supplementary Table S4.

### 3.8. Results of Certainty of Evidence

The GRADE assessment demonstrated that the certainty of evidence for all evaluated outcomes was very low. The prevalence of *Salmonella* serovars across countries and sources (food production environment and clinical isolates) was assessed using 93 studies, while ARG prevalence and specific resistance determinants such as β-lactamase, tetracycline, aminoglycoside, quinolone, sulfonamide, chloramphenicol resistance, and MDR were evaluated across 8–57 studies. Although most studies exhibited low to moderate risk of bias and there were no serious concerns regarding indirectness or imprecision, all outcomes showed serious inconsistency due to extremely high heterogeneity (I^2^ ranging from 96.95% to 99.92%), indicating substantial variability in prevalence estimates across studies (Table 7).

## 4. Discussion

In this systematic review and meta-analysis, we aim to demonstrate the evidence of the genetic similarity of ARGs in *Salmonella* isolates from food production environments and clinical settings in the ME region. Our report demonstrates both important awareness and important research gaps.

Our analysis included 94 studies covering varied geographic areas and source types, demonstrating that specific classes of resistance and AMR are common in both food production environments and clinical settings. This aligns with the findings of another review, which estimated *Salmonella* prevalence at 6.6% (95% CI, 5.4 to 7.9%) in ME and North Africa [8]. Similarly, in the Chinese egg retail market, the prevalence of *Salmonella* was 7.20%, which is higher than in Europe and the United States [122]. The AMR rate in China was 73.63% (95% CI, 0.68–0.99) [123]. Furthermore, AMR, particularly carbapenem and ESBL-producing organisms in *Salmonella*, was also observed in the Pakistani pediatric population [124]. Our meta-analytical outcomes indicated a significant prevalence of *Salmonella* isolates in ME regions, and that resistance genes for multiple antibiotic classes are common in both environments. β-lactamases, tetracycline, aminoglycoside, sulfonamide, quinolone, chloramphenicol resistance genes and MDR are commonly found in isolates from both sources, suggesting that resistance is deeply rooted in the regional *Salmonella* isolates. However, due to high heterogeneity, the certainty of evidence was very low; therefore, the interpretation of these outcomes should be done with caution. Meanwhile, this high heterogeneity is expected due to genuine clinical and methodological diversity across the included studies, including differences in the characteristics of samples (animal, environmental, and human), disease severity, specimen’s collection approaches, protocols for laboratory assays, and biomarker quantification techniques. Additionally, differences in healthcare and surveillance systems and variation in local antimicrobial use and resistance patterns may also contribute to this high heterogeneity among the included studies.

On the other hand, studies from the ME region demonstrate the public health significance of foodborne diseases. However, there was a geographical bias, which means there were data limitations from other ME countries, including Iraq, Syria, Yemen, and Palestine. Most studies were reported from Egypt and Iran. This may be due to the political instability and resource limitations in some of these countries, which make research difficult, but they may also create an unchecked environment for AMR to flourish. The temporal trend we reported, with most studies published in the last 10 years, reflects the increased adoption of molecular and WGS methods in the region. This is promising because WGS provides the method needed to track back transmission and identify genetic relationships. However, even recent WGS studies have not applied direct comparative analyses between food production environments and clinical isolates, suggesting that the research questions are not fully answered with the technological capabilities now available. This demonstrates an essential knowledge gap that prevents us from drawing strong conclusions about transmission pathways between these two environments.

The STs we reported from food production environments and clinical settings (ST19 and ST198) suggest potential transmission links. These conclusions align with what we know from other regions, where food to human transmission of *Salmonella* has been documented [9,11]. However, the prevalence of the same ST in both sources does not necessarily provide evidence of transmission, these could reflect independent introductions of the same clone, or transmission could be happening in the opposite direction (human to food) or through environmental factors. Lacking direct genomic comparisons showing high genetic similarity, we basically cannot establish these connections with confidence. Therefore, the presence of shared STs was not interpreted as definitive evidence of transmission or epidemiological linkage. Even then, these shared STs suggested that improved surveillance systems monitoring both food production environments and clinical settings at the same time would be important and helpful. These surveillance efforts could help in detecting resistance patterns early and identifying transmission pathways. The success of PulseNet and Genome Trakr demonstrates the importance of this approach [18,125,126,127].

Interestingly, the overall ESBL gene distribution has been shown in this study. Food production environment isolates showed a higher prevalence of these genes, including *bla_TEM_*, *bla_CTXM_*, and *bla_CMY_* (50%, 48%, and 42%) for animal source, animal and human source, and animal, human, and environment sources, respectively, compared to the clinical isolates (36%). Our findings align with another meta-analysis, which also showed a high prevalence of ESBL genes in animal isolates (31%, 95% CI, 17 to 29%), and 26% (95% CI, 20 to 34%) in human isolates [128]. However, there were contrasting outcomes in the distribution at the gene level: we observed a high prevalence of bla_TEM_ (64%), while another meta-analysis observed a high prevalence of Salmonella (25.20%) in collected samples. Additionally, blaCTXM15 genes associated with Enterobacterioceae, including *Salmonella*, were found in 61% of human isolates and 85% of animal isolates [128]. This may be due to many causes: it may indicate that *Salmonella* isolates are acquiring ESBL genes through gene transfer within the food production environment, where humans are also working. Another reason may be the use of high levels of antibiotics, particularly in the poultry industry. This pattern may simply reflect the differences in the serovars and STs that dominate in each environment with food production environment isolates more likely to belong to clones that carry ESBL genes. Carbapenem resistance in *Salmonella* is still rare worldwide [87] and its emergence in the ME could be problematic. Carbapenems are critical antibiotics for treating serious infections and resistance to such a drug will limit treatment options.

Furthermore, other classes, like tetracyclines, aminoglycosides, quinolones, sulfonamides, and chloramphenicol, and their gene variants *tetA*, *aadA2*, *qnrS*, *sul1*, and *floR*, also demonstrated high prevalence (65%, 99%, 50%, 65%, and 81%) in food production environments and clinical isolates. Meanwhile, in human isolates, a high overall prevalence of quinolone and sulfonamide resistance was observed. This aligns with findings from another meta-analysis in China, which found AMR in quinolones and β-lactums (49.51% and 73.76%) [129]. This high prevalence of these ARGs highlights a major “One Health” concern regarding the spread of AMR from both the food production environment and clinical isolates [130]. The resistance genes we identified are aligned with global trends, as *bla_CTXM_*, *bla_CMY2_*, *tet* genes, and aminoglycoside resistance are common in *Salmonella* worldwide [1,131]. This high prevalence also underlines the selective pressure and persistence of these genotypes across the food production environment and food chain [132]. Meanwhile, co-detection of these classes, particularly ESBLs and quinolones, in food production environment and clinical isolates poses a formidable challenge for effective antimicrobial therapy [133,134].

Moreover, a high MDR rate (66%) for ampicillin and ciprofloxacin was observed in the ME region. These MDR isolates were also reported from Korea, which are identical to those isolates from Indonesia and India [135]. Alarmingly, resistance to last-resort antibiotics, including colistin, imipenem, and meropenem, was also identified in some studies, although at lower frequencies, indicating the emergence of highly resistant lineages. Moreover, the emergence of mobile colistin resistance genes has been described as the most serious scenario for public health [136]. The presence of mobile colistin resistance in the food production chain represents a worrying sign that needs immediate surveillance and intervention efforts. Surveillance is very important, as *Salmonella* infected patients had longer hospital stays (6.4 days, 95% CI, 4.9–7.8) for susceptible cases, and for resistant cases, it was 8.4 days (95% CI, 5.1–11.7), and it also increased healthcare costs [137]. In China, the case fatality rate of invasive non-typhoidal *Salmonella* was 8.6% (95% CI, 6.8–10.4), which further emphasizes the integrated approach to surveillance [138].

Our findings highlighted why One Health approaches are important for addressing AMR. The resistance genes and STs we documented are not limited to specific environments, including food production environments and clinical settings. Notably, overlap in PCR prevalence, resistance patterns, and STs between sources should not be interpreted as evidence of transmission, establishing transmission requires contemporaneous, epidemiologically linked sampling followed by high-resolution WGS and cgMLST or SNP-based phylogenetic analysis. The IncI1 plasmids carrying bla_CMY2_ that we observe narratively in both food production environments and clinical isolates demonstrate how resistance can transfer through ecological environments. The class 1 integrons with identical gene cassette arrays appearing in different serovars show that horizontal gene transfer is actively happening in *Salmonella* populations.

Yet, even with this clear relation, our review demonstrates that research efforts remain separated or isolated. Food production environment studies and clinical studies are typically conducted by different research groups, published in different journals and rarely integrated. This scattering of research efforts reflects broader challenges in implementing One Health approaches; institutional barriers, funding structures, and disciplinary boundaries often prevent the integrated investigations needed.

The high prevalence of resistance genes in food production environments strongly suggests more restrictions on antibiotic use in agriculture. It is worth mentioning that many ME countries have started developing national action plans on AMR, yet the implementation of such action plans has been variable. Our findings provide evidence to support policies controlling the use of antibiotics (especially colistin and third-generation cephalosporins) in the food production environment.

### Limitations

This systematic review has many limitations that readers should keep in mind when interpreting the findings.

**Evidence gap**: The most notable limitation in this review is the absence of direct comparative genomic studies between food production environments and clinical isolates from the same geographical areas. The gap indicates that many of our conclusions about potential transmission pathways must remain speculative. We can describe which resistance genes and STs are present in each source, yet, we cannot establish genetic similarity or transmission direction.
**Diversity in methods and reporting**: The included studies use diverse molecular methods, sampling strategies, and reporting standards. While we try to consider these differences in our synthesis, it is unavoidable that uncertainty is introduced. For example, PCR-based studies that test for specific genes may miss other genes, while WGS studies provide more comprehensive data, but they are not always analyzed consistently. In addition, some studies reported complete plasmid sequences while others only identified replicon types, making it difficult to assess whether similar plasmids are truly identical.
**Geographic coverage**: This systematic review is more oriented towards the ME region, especially Egypt and Iran. The studies span a decade (2015–2026) during which AMR patterns have evolved. Although we tried to investigate temporal trends, the limited number of studies from each country and time period made reliable trend analyses difficult.
**Publication bias**: We cannot exclude publication bias (studies finding no resistance or no interesting patterns may be less likely to be published). In addition, several studies may have collected data but did not report it if it was not their main focus. This selective reporting could bias our prevalence estimation.
**Language limitations**: By limiting our search to English publications, we may have missed related studies published in other languages, such as Persian. However, most publications are in English, so we believe this limitation is minor.
**Lack of patient data**: Many clinical studies did not report patient information (travel history, food consumption patterns, and antibiotic use), which would have been important to understand the risk factors. Similarly, food production studies did not provide information about antibiotic use on farms, making it difficult to link resistance patterns to specific interventions.
**Risk of bias**: As mentioned earlier, many studies had methodological limitations (incomplete quality control reporting and lack of statistical analysis). While we used the GRADE methodology to account for these limitations in our certainty assessments, they affected the reliability of our conclusions.
**High heterogeneity:** All of the meta-analytic outcomes demonstrated high heterogeneity, which can limit the interpretation of the findings. Meanwhile, τ^2^ and prediction intervals were not directly available within the analytical framework of OpenMeta [Analyst].

## 5. Conclusions

This systematic review demonstrates the real complexity of our understanding of *Salmonella* antibiotic resistance in ME. On one hand, we have more and more molecular data documenting the prevalence and diversity of resistance genes in both food production environments and clinical settings. On the other hand, direct comparative evidence is needed to establish whether and how resistant strains transfer between both environments. Resistance genes to antibiotics are widespread in *Salmonella* in both food and clinical environments in ME countries. β-lactamases, tetracycline, quinolone, aminoglycoside, and chloramphenicol resistance genes are common in both environments. Several STs (ST19, ST198) were found in both food production and clinical isolates within many countries. Although molecular evidence suggested the occurrence of shared STs across some reservoirs, direct comparative genomic evidence sufficient to establish transmission pathways was largely unavailable. Therefore, we cannot establish genetic similarity between food production and clinical *Salmonella* in the ME. We do not know whether the shared STs we detected represent actual transmission events or independent introductions of the same clones. Moreover, we cannot conclude the direction of transmission whether resistant strains are moving from food production to humans or from humans to food environments, or through more complex ways, including environmental reservoirs. There is an urgent need for research investigation that directly compares *Salmonella* isolates from both food and clinical environments from the same ME geographic areas using WGS and comparative genomic methods. Studies investigating the effect of intervention methods to reduce antibiotic use in food animal production and their impact on AMR in both environments *Salmonella* isolates would provide valuable evidence for policy improvement. Furthermore, future studies must adopt the One Health surveillance approach in terms of simultaneous sampling from different sources, including humans, animals, food, and environments. This should be followed by WGS analysis of the recovered Salmonella isolates to assess genetic similarity among isolates. Such analysis will allow the detection of shared lineages, AMR markers, and virulence genes. When WGS analysis is combined with epidemiological and source metadata, it can help in the identification of potential transmission links and reservoirs across different domains. Importantly, the standardization of longitudinal sampling across interconnected One Health compartments will allow direct comparison of isolates from the same place and time to overcome fragmented sampling and non-comparable genomic data.

## Figures and Tables

**Table 2:** Summary of the general characteristics of molecular methods and resistance genes found in antibiotics used for the management of *Salmonella* infections.

Study ID	Molecular Method Type	WGS Used	Specific Resistance Classes Detected	Gene Variants
Food production environment				
[53]	Multiplex PCR	No	Aminoglycoside, Dihydrofolate, β-lactamase, Florfenicol, Tetracyclines	*aadA1*, *aadA2*, *aadA5*, *dfrA1*, *dfrA5*, *dfrA12*, *dfrA15*, *dfrA17*, *bla_TEM1_*, *bla_CMY2_*, *floR*, *tetA*
[40]	PCR	No	β-lactamase, Quinolone, Florfenicol	*aadA2*, *bla_OXA1_*, *bla_TEM1_*, *bla_CMY2_*, *bla_CTXM3_*, *bla_SHV12_*, *qnrA*, *qnrB*, *qnrS*, *aac(6′)-Ib-cr*, *floR*
[54]	PCR	No	ESBL, Sulfonamides	*bla_TEM1_*, *bla_SHV1_*, *sul1*, *sul2*
[55]	PCR	No	NA	NA
[56]	PCR and gel electrophoresis	No	β-lactamase	*bla_TEM1_*
[57]	PCR and gel electrophoresis	No	NA	NA
[58]	PCR	No	Tetracyclines, Sulfonamides	*gyrA*, *sul1*, *tetA*
[59]	PCR	No	β-lactamase, Cephalosporins, Chloramphenicol, Tetracycline, Sulfonamides	*bla_TEM_*, *aadB*, *aadC*, *aadA1*, *aadA2*, *floR*, *tetA*, *tetA(B)*, *sul1*
[60]	PCR	No	β-lactamase	*bla_TEM_*
[41]	ERIC-PCR and (GTG)5	No	NA	NA
[61]	PCR and gel electrophoresis	No	Quinolone	*qnrA*, *qnrB*, *qnrS*, *qepA*
[42]	PCR	No	ESBL, AmpC β-lactamase, Quinolone	*bla_CTXM_*, *bla_TEM1_*, *bla_CMY_*, *qnrA*, *qnrB*, *qnrS*
[62]	PCR	No	AmpC β-lactamase, Tetracyclines, Sulfamethoxazole	*bla_TEM_*, *tetA*, *tetC*, *sul1*, *sul3*, *cat1*, *floR*
[63]	PCR	No	Tetracyclines, Chloramphenicol	*tetA*, *tetB*, *cat1*, *floR*, *strA*
[64]	PCR	Yes	β-lactamase, Tetracyclines, Chloramphenicol, Sulfonamides	*sul1*, *bla_CARB2_*, *bla_DHA1_*, *bla_CMY2_*, *bla_TEM1B_*, *floR*, *dfrA14*, *erm(42)*, *aph(3′) Ia*, *aadA1*, *aadA2*, *aadA7*, *strA*, *strB*, *tetG*, *tetA*, *tetH*, *dfrA14*, *erm(42)*, *aph(3′) Ia*, *sul1*, *sul2*
[43]	PCR	No	β-lactamase, Tetracyclines, Chloramphenicol	*bla_TEM_*, *tetA(A)*, *floR*
[65]	PCR	No	β-lactamase, Quinolone	*qnrS*, *qnrA*, *bla_TEM_*
[66]	PCR	No	β-lactamase, Quinolone	NA
[67]	PCR	No	β-lactamase, Quinolone, Aminoglycosides	*bla_TEM_*, *aac3-Id*, *aadA7*, *qnrS*
[68]	PCR	No	Tetracycline	*tetA*
[69]	PCR, MLST	Yes	Aminoglycosides, Tetracyclines, Sulfonamides	*bla_TEM1B_*, *bla_CMY2_*, *aadA7*, *aac(3)-Id*, *tet(A)*, *sul1*
[70]	Multiplex PCR	No	ESBL	*bla_TEM_*, *bla_SHV_*
[52]	Multiplex PCR	No	NA	NA
[47]	PCR	No	β-lactamase	*bla_CTXM3_*, *bla_CTXM14_*, *bla_CTXM15_*, *bla_SHV1_*, *bla_SHV2_*, *bla_SHV12_*
[71]	PCR	No	Quinolone, β-lactamase, Tetracyclines	*bla_TEM_*, *tetA(A)*, *qnrS*
[72]	Multiplex PCR and gel electrophoresis	No	β-lactamase	*bla_CMY1_*, *bla_CMY2_*, *bla_OXA2_*
[73]	PCR	No	Aminopenicillins, Aminoglycosides, Polypeptide ABC’s, Florfenicol, Tetracyclines, Fluoroquinolones	Not detected
[44]	PCR	No	β-lactamase, PMQR	*bla_CTXM1_*, *bla_CTXM3_*, *bla_CTXM13_*, *bla_CTXM14_*, *bla_CTXM15_*, *bla_SHV12_*, *bla_CMY2_*, *bla_TEM1_*, *bla_OXA1_*, *qnrS*, *qnrA*, *qnrB*, *aac(6′)-Ib-cr*
[74]	PCR	No	β-lactamase, Tetracycline, Trimethoprim-sulfamethoxazole, Sulfonamides	*bla_IMP_*, *bla_OXA48_*, *bla_NDM_*, *tetA*, *tetB*, *dfrA1*, *sul1*
[75]	PCR	No	ESBL, PMQR	*blas_TEM_*, *qnrB*
[76]	ERIC-PCR	No	Aminoglycosides, Tetracyclines, Folate pathway inhibitors, Sulfonamides, Macrolides, Phenicol, β-lactamase	*aadA1*, *tetA*, *dfrA1*, *aac(3)-IV*, *sul1*, *bla_TEM_*
[51]	PCR, MLST	Yes	Sulfonamides, β-lactamase, Tetracyclines, Quinolone	*(mcr)-1.1*, *sul2*, *tetA*, *bla_TEM1B_*, *qnrB19*, *aac(6′)-Iaa*, *aac(3)-IV*, *and floR*, *sul2*, *tetA*, *bla_TEM1B_*, *qnrB19*, *aac(6′)-Iaa*
[77]	PCR	No	Tetracyclines, florfenicol	NA
[78]	PCR and gel electrophoresis	No	β-lactamase	*bla_TEM_*, *bla_SHV_*
[79]	PCR	No	ESBL, Tetracyclines	*bla_TEM_*, *tetA*
[45]	ERIC-PCR and gel electrophoresis	No	β-lactamase, Tetracycline, Aminoglycoside, Sulfonamide, Cephalosporins, Macrolides, Fluoroquinoloes, Sulphonamides, penicillin, Phenicols	*bla_TEM_*
[80]	PCR	No	Tetracyclines	*tetA*, *tetB*
[81]	PCR, MLST	Yes	β-lactamase, Aminoglycosides, Phenicols, Tetracycline, Trimethoprim, and Sulfonamides, Lincosamide, Quinolones, Macrolides	*aac (3)-IV*, *aph(4)-Ia*, *ant(3″)-Ia*, *sul1*, *sul2*, *dfrA14*, *tetA*, *tetD*, *bla_CMY2_*, *bla*_TEM1D_, *bla_CTXM65_*, *bla*_TEM1B_, *qnrB19*, *qnrB81*, *qnrS1*, *floR*, *erm(42)*
[82]	PCR	No	B-lactamase, Tetracycline, Aminoglycoside, Sulfonamide, Macrolide	*bla_TEM_*, *bla_CTXM_*, *bla_NDM_*, *ereA*, *sul1*, *aadA1*, *tetA*
[83]	PCR	No	ESBL, Sulfonamide	*bla_TEM_*, *sul1*
[84]	PCR	Yes	Tetracycline, Aminoglycosides, β-lactamase, Quinolone	*aac(6′)-Iaa*, *aac93)Via*, *tet(A)*, *sul1*, *sul2*, *fosA7*, *bla_CMY2_*, *bla_TEM1B_*, *mphB*, *qnrB19*
[85]	PCR	Yes	Aminoglycoside, β-lactamase, Quinolone, Sulfonamide	*aadA1*, *bla_CMY2_*, *bla_SHV12_*, *qnrB19*, *qnrS1*, *Sul1*, *Sul2*
[86]	PCR	No	ESBL	*bla_TEM_*, *bla_SHV_*, *bla_CTXM_*, *bla_CMY_*
[87]	PCR	No	β-lactamase	*bla_TEM_*, *bla_NDM1_*, *bla_DHA_*, *bla_CTXM_*
[88]	Multiplex PCR	No	β-lactamase	*bla_OXA_*, *bla_CTXM1_*, *bla_TEM_*
[89]	PCR	No	ESBL, Quinolone	*qnrS*, *bla_TEM_*, *bla_SHV_*
[90]	PCR	No	β-lactamase	*bla_TEM_*, *bla_SHV_*, *bla_CTXM_*
[91]	Multiplex PCR	No	β-lactamase, Tetracycline, Aminoglycoside, Sulfonamide	*bla_TEM_*, *bla_OXA_*, *bla_SHV_*, *sul1*, *aadA*, *dhfrv*, *tetK*, *tetA*
[92]	PCR	No	Penicillins, Cephalosporins, Tetracyclines, Phenicol, Fluoroquinolones, Quinolones, Aminoglycosides, Folate-pathway inhibitors	*bla_TEM_*, *bla_OXA_*, *catAI*, *floR*, *tet(A–D*, *G*, *E)*, *strA*, *strB*, *aadA*, *aac-3-Iia*, *aph-3-Iia*, *aph-3-1*, *aph-3-Ia*
[93]	PCR	No	Furazolidone	*nfsA*, *nfsB*
[94]	PCR	No	β-lactamase, Sulfonamide	*bla_TEM_*, *tetB*, *sul1*, *sul2*
[95]	PCR	Yes	Tetracyclines, Aminoglycoside, Sulfonamide, Colistin	*mcr1.1*, *aadA1*, *sul1*, *tetA*, *qacEdelta1*
[96]	Multiplex PCR	No	β-lactamase, Tetracycline, Sulfonamide, Streptomycin	*bla_TEM_*, *tetA*, *tetB*, *sul1*, *strA/B*
[46]	PCR	No	β-lactamase, Tetracycline, Sulfonamide, Streptomycin	*bla_TEM_*, *tetA*, *sul1*, *Sul2*, *Sul3*, *StrA/B*
[97]	Uniplex PCR	No	ESBL, Cholistin	*bla_TEM_*, *bla_OXA10_*, *bla_SHV_*, *bla_CTXM_*, *bla_CMY2_*, *bla_OXA2_*, *mcr1*
[98]	RT-PCR	Yes	β-lactamase, Cephalosporins, Fluoroquinolones, Tetracyclines, Aminoglycoside, Sulfonamides	*qnrB19*, *dfrA14*, *aph(6)-Id*, *aph(3″)-Ib*, *aph(3″)-Ia*, *qnrS1*, *bla_CMY2_*, *sul2*, *tetA*
[99]	PCR	Yes	Quinolone, β-lactamase, Aminoglycoside, Sulfonamide, trimethoprim, Tetracycline	*qnrB19*, *qnrB*, *qnrA1*, *bla_CMY2_*, *bla_TEM_*, *bla_CTXM55_*, *aac(3)-IVa*, *aadA2*, *aadA22*, *ant(2″)-Ia and aph (3′)-Ia*, *sul1*, *sul2*, *tetA*, *tetD*
[100]	PCR	Yes	Aminoglycosides, β-lactamase, Tetracyclines, Polymyxins, Florfenicol, Macrolides, Fluoroquinolones, Sulfonamides	*aac6′*, *aac3*, *ant3″*, *aph3′*, *aadA*, *aph4)*, *bla_TEM_*, *bla_CTXM_*, *bla_CMY2_*, *tetA*, *tetB*, *tetD*, *mcr1*, *mcr9*, *floR and cmlA*, *cat*, *lnuF*, *mphA*, *qnrB*, *sul*
[101]	PCR	No	β-lactamase, Colistin	*bla_DHA_*, *mcr-1*
[102]	PCR	No	β-lactamase, Tetracyclines	*tetA*, *bla_TEM_*
[103]	PCR	No	ESBL	*bla_TEM_*, *bla_SHV_*, *bla_CTXM_*
[104]	PCR and gel electrophoresis	No	NA	*aac(3)-Id*, *aadA2*, *aadA4*, *aadA7*, *dfrA15*
[105]	PCR	No	β-lactams, fluoroquinolones, aminoglycosides, and tetracyclines	NA
[106]	PCR	No	Aminoglycosides	NA
[107]	PCR	No	ESBL	*bla_TEM_*
[108]	Uniplex PCR	No	NA	NA
[109]	PCR	No	Tetracyclines	tetA, tet(X4)
[110]	PCR	No	β-lactamase, PMQR	bla_CMY2_, bla_TEM_
[111]	PCR	No	ESBL, Quinolone	bla_TEM_, int1, bla_CTX_, qnrS, int3
[112]	Multiplex PCR	No	NA	NA
[113]	PCR	No	ESBL, Tetracyclines, Cephalosporins	bla_TEM_, tetA, floR
[114]	PCR	No	ESBL	bla_TEM_, bla_SHV_, bla_CMY2_
[115]	PCR, MLST	Yes	Aminoglycoside, Trimethoprim, β-lactamase, Sulfonamide, Tetracycline	AAC(6′)-Iy, ANT(3″)-Ia, dfrA1, bla_TEM1_, floR, sul2, tetA
[116]	PCR	No	β-lactamase	bla_OXA2_, bla_CMY1_
[117]	Multiplex PCR	No	Aminoglycosides, β-lactamase, Cephalosporins, Tetracycline	tetA, sul1, aadA1, aac(3)-IV
[118]	PCR and gel electrophoresis	No	Aminoglycoside, quinolones	aadA-2, qnrA
[119]	PCR	No	β-lactamase, Trimethoprim–Sulfamethoxazole, Chloramphenicol, Tetracycline, Fluoroquinolone	bla_CARB_, dfrA1, floR, tetA, *gyrA*, *parC*
[120]	PFGE	No	NA	NA
[121]	PCR	No	Aminoglycosides, β-lactamase, Tetracycline, Cholistin	aadA, bla_TEM_, blaZ, tetA, dfrA, sul1, mcr1
[48]	PCR	Yes	Aminoglycosides, Fosfomycin	aac(6′)-Iaa, fosA7
[49]	PCR	No	ESBLs	bla_CTXM_
[50]	PCR	No	Penicillin, β-lactamase, Aminoglycosides, Fluoroquinolones, Tetracyclines, Folate pathway antagonists	NA
Clinical isolates				
[29]	PCR and PFGE	No	β-lactamase, Fluoroquinolones, Aminoglycosides, Tetracyclines, Folate pathway inhibitors	NA
[31]	PCR	No	ESBL	bla_CTXM15_, bla_TEM_
[30]	PCR	No	Penicillins, Aminoglycosides, Quinolones, Tetracyclines, Sulphonamides, Polymyxin, Phenicols, Lincosamides	NA
[32]	PCR	No	β-lactamase	bla_CTXM15_, bla_TEM169_, bla_PER1_
[28]	PR and PFGE	No	β-lactamase, Aminoglycosides, Tetracyclines, Quinolones, Sulphonamides, Chloramphenicol	NA
[39]	PCR	No	ESBLs	bla_CTX_, bla_TEM_, bla_SHV_
[35]	PCR	No	β-lactamase, Aminoglycosides, Fluoroquinolones, Tetracyclines, Sulfonamides/Folate Pathway Antagonists,	bla_TEM1_, bla_PSE1_
[33]	PCR	No	Sulfonamide, Fluoroquinolone, Integrase, Quinolone	sul1, sul2, gyrA, parC, qnrS, qnrA, qnrB, int1, int2, int3
[38]	PCR	No	Quinolone	qnrS
[37]	PCR	Yes	Aminoglycoside, Quinolones, ESBL, Sulfonamide	qnr S1, bla_CTXM15_, aph(6)-Id, aph(3″)Id, aac(6′)-Iaa, sul1, sul2, dfrA7
[34]	PCR, MLST	Yes	β-lactamase, Aminoglycosides, Fluoroquinolones, Tetracyclines, Sulfonamides/Folate Pathway Antagonists,	NA
[36]	RT-PCR	No	Tetracyclines, Chloramphenicol, Cephalosporins, Quinolones, Colistin	pmrA, pmrB, iC, agfA

Abbreviations: PCR = Polymerase chain reaction; ESBL = Extended-spectrum beta-lactamase; NA = Not applicable; MLST = Multilocus sequence typing; ERIC = Enterobacterial repetitive intergenic consensus; RT = Reverse transcription.

**Table 3:** Summary of genotyping outcomes.

Study ID	Location (Chromosomal vs. Plasmid)	Mobile Genetic Elements (Integrons, Transposons, Plasmid Type)	Sequence Types
Food production environment			
[53]	NA	Integrons class 1 and 2	NA
[40]	Plasmid and chromosomal	Integrons class 1 and 2	NA
[104]	Plasmid and chromosomal	Integron (class 1)	NA
[56]	Plasmid	Plasmid type	NA
[41]	Plasmid	Plasmid type	NA
[57]	Plasmid and chromosomal	*hil*A, *sop*B, *stn*, *pef*A, and *spv*C genes	NA
[59]	NA	Integrons (class 1)	NA
[60]	NA	Integrone (class 1)	NA
[61]	Plasmid and chromosomal	NA	NA
[63]	NA	Integrons (class 1, 2, 3)	NA
[64]	NA	NA	ST11, ST15, ST19, ST198, ST413
[65]	Plasmid	NA	NA
[67]	NA	Integron (class 1)	NA
[107]	NA	Integrons	NA
[69]	Plasmid and chromosomal	NA	ST198
[108]	Plasmid and chromosomal	NA	NA
[52]	Plasmid	spvC	NA
[47]	Plasmid	IncI1, IncA/C, IncHI1, IncN	SE-T1-T3, SE-M1, ST-T1, ST-T2, ST-M1, SK-T1, SK-T2, SK-M1
[44]	Plasmid	NA	NA
[75]	Plasmid	NA	NA
[76]	Plasmid	Plasmid type	NA
[51]	Plasmid	Col(pHAD28), IncC, IncFII(S), IncI1-I(Alpha).	ST548
[110]	Plasmid	NA	NA
[45]	NA	Integron (class 1)	NA
[49]	Plasmid	NA	NA
[48]	NA	NA	ST-novel, ST463, ST3760, ST64
[81]	Plasmid	IncA/C2, IncFIB(K)_1_Kpn3, Col440I_1, IncR, IncX1, IncI1_1_Alpha, IncFIB(S)/IncFII(S), IncHI2/IncHI2A, IncX2, ColpVC	ST548, ST32, ST11, ST198
[115]	Plasmid	NA	NA
[82]	Plasmid	Plasmid type and integrons type	SE20, ST15
[84]	Plasmid	IncC, Col(pHAD28)	ST15, ST152, ST198, ST548
[85]	Plasmid	IncFIB	ST32, ST458, ST198, ST2133, ST543
[87]	Plasmid	NA	NA
[89]	Plasmid	NA	NA
[91]	Plasmid	spvB, spvC, spvR	NA
[92]	NA	Integrons (class 1, 2, 3)	NA
[95]	Plasmid	IncX4, IncFIB	ST32
[97]	Plasmid	Integrons (class 1)	NA
[117]	Plasmid	NA	NA
[98]	Plasmid	IncFIB(K)_1_Kpn3	ST11, ST19, ST15
[99]	Plasmid	Plasmid mediated	ST22
[100]	Plasmid and chromosomal	IncC	NA
[118]	Plasmid	Plasmid mediated	NA
[101]	Plasmid	NA	NA
[102]	NA	Integrons (class 1)	NA
Clinical isolates			
[30]	NA	Integron (class 1)	NA
[32]	Plasmid	Plasmid mediated	NA
[35]	Plasmid and chromosomal	NA	NA
[33]	Plasmid	Integron (class 1, 2), Plasmid mediated	NA
[38]	Plasmid	Plasmid mediated	NA
[37]	NA	NA	ST1, ST10
[34]	NA	NA	ST58, ST183, ST1918, ST19, ST29, ST198, ST158, ST166

Abbreviations: NA = not applicable.

**Table 4:** Summary of phenotypic resistance status against antibiotics used in GCC countries.

Study ID	Drug Resistance Status
Food production environment isolates	
[53]	Ampicillin = 20/21, streptomycin = 20/21, spectinomycin = 18/21, kanamycin = 18/21, tetracycline = 18/21, chloramphenicol = 17/21, trimethoprim/sulfamethoxazole = 16/21, ciprofloxacin = 10/21, nalidixic acid = 15/21
[40]	Ampicillin (95.7%), then to kanamycin (93.6%), spectinomycin (93.6%), streptomycin (91.5%) and sulfamethoxazole/trimethoprim (91.5%).
[54]	Sulfamethoxazole = 76/78, nalidixic acid = 75/78, tetracycline = 75/78, ampicillin = 59/78, chloramphenicol = 44/78
[104]	Erythromycin and tetracycline = 12/12, amoxicillin-clavulanic acid = 11/12, trimethoprim-sulfamethoxazole = 10/12, streptomycin = 9/12, nalidixic acid = 9/12, ampicillin-sulbactam = 9/12, gentamycin = 8/12, ampicillin = 8/12, chloramphenicol = 7/12, ciprofloxacin = 3/12, ceftriaxone = 3/12
[55]	Erythromycin = 68/68, penicillin = 68/38, amoxicillin = 68/68, nalidixic acid = 67/68, sulphamethoxazole = 66/68, oxytetracycline = 65/68, ampicillin = 62/68
[56]	Ampicillin = 65/100, Amoxicillin = 68/100, Ticarcillin = 88/100, Cefazolin = 100/100
[41]	The highest resistance (96.7%) was observed to nitrofurantoin
[57]	Amoxicillin/clavulanic acid, streptomycin = 5/17, gentamicin = 2/17, ceftriaxone = 1/17, sulfamethoxazole/trimethoprim = 6/17, nalidixic acid = 9/17, doxycycline = 7/17, chloramphenicol = 3/17, colistin sulfate = 3/17
[58]	Amoxicillin = 2/6, ampicillin = 2/6, neomycin = 2/6, enrofloxacin = 1/6, ciprofloxacin = 1/6, erythromycin = 1/6
[59]	Chloramphenicol = 61/78, trimethoprim–sulfamethoxazole = 61/78, streptomycin = 44/78, tetracycline = 41/78, ampicillin = 41/78, gentamicin = 23/78
[60]	Vancomycin = 23/23, oxacillin = 21/23, amoxicillin = 18/23, erythromycin = 18/23, nalidixic acid = 18/23
[119]	Erythromycin = 33/33, cefalotin, cefuroxime = 30/33, cefuroximeaxetil = 30/33, cefoxitin = 29/33, gentamicin = 30/33, amikacin and tobramycin = 29/33, Nitrofurantoin = 19/33, streptomycin = 9/33, tetracycline = 8/33, trimethoprim–sulfamethoxazole = 6/33, neomycin = 5/33
[120]	Clinical: Ampicillin = 68/290, trimethoprim–sulfamethoxazol = 23/290, ciprofloxacin = 11/290, ceftazidime = 7/290; Food isolates: Ampicillin = 10/49, ciprofloxacin = 5/49, ceftazidime = 1/49
[61]	Norfloxacin = 81/81, ciprofloxacin = 46/81, enrofloxacin = 18/81, nalidixic acid = 30/81
[42]	Amoxicillin/clavulanate = 8/15, Ampicillin = 13/15, cefotaxime = 12/15, cefpodoxime = 9/15, ceftazidime = 4/15, ceftriaxone = 5/15, enrofloxacin = 1/15, nalidixic acid = 5/15, streptomycin = 4/15, tetracycline = 6/15, trimethoprim/sulphamethoxazole = 8/15
[62]	Ampicillin = 58/58, chloramphenicol = 58/58, trimethoprim-sulfamethoxazole = 3/58, tetracycline = 58/58
[63]	Nalidixic acid and trimethoprim resistant
[64]	Ampicillin = 7/46, amoxicillin/clavulanate = 3/46, cefotaxime = 8/46, ceftriaxone = 6/46, gentamicin = 7/46, streptomycin = 32/46, chloramphenicol = 2/46, ciprofloxacin = 10/46, tetracycline = 39/46, azithromycin = 6/46, nalidixic acid = 37/46, trimethoprim = 30/46
[105]	Ciprofloxacin = 40/44, nalidixic acid = 32/44 and cefuroxime = 32/44
[43]	Amoxicillin-clavulanic acid = 2/2, cefotaxime = 1/2, chloramphenicol = 2/2, clindamycin = 1/2, doxycycline = 2/2, erythromycin = 1/2, gentamicin = 1/2, methicillin = 2/2
[65]	cefuroxime (100%), nalidixic acid (93%) and amoxicillin/clavulanic acid (83%), cefepime (53%), streptomycin (40%), sulfamethaxezole/trimethoprim (40%), ampicillin (37%), doxycycline (37%) and 30% to gentamicin
[66]	Trimethoprim-sulfamethoxazole = 120/120, amoxicillin-clavulanic acid = 82/120, ampicillin = 82/120, streptomycin = 78/120
[106]	Amikacin = 16/16, gentamicin = 15/16, tobramycin = 14/16
[67]	Gentamicin = 5/10, ciprofloxacin = 5/10, spectinomycin = 5/10, colistin = 5/10
[68]	Ampicillin10/17, chloramphenicol = 3/17, ciprofloxacin = 4/17, gentamicin = 7/17, nalidixic acid = 6/17, nitrofurantoin = 6/17, norfloxacin = 6/17, trimethoprim-sulfamethoxazole = 8/17, tetracycline = 12/17, levofloxacin = 3/17, ceftriaxone = 4/17
[107]	Chicken meat samples: Tetracycline: 5/75, trimethoprim/sulfamethoxazole = 1/75; Stool samples: Tetracycline = 1/22, azithromycin = 1/22
[69]	Ciprofloxacin
[108]	Azithromycin = 18/31, cephalexin = 29/31, cefuroxime = 26/31, cefaclor = 27/31, erythromycin = 30/31, sulfamethoxazole/ trimethoprim = 29/31, streptomycin = 25/31, neomycin = 5/31, vancomycin = 26/31, norfloxacin = 0/31, doxycycline = 29/31, penicillin = 22/31, gentamycin = 1/31, tetracycline = 26/31, amoxicillin = 22/31, amoxicillin/clavulanic acid = 26/31, rifampin = 22/31
[70]	Cefpodoxime = 18/20, cefotaxime = 16/20, ceftazidime = 9/20, ceftriaxone = 8/20, aztreonam = 8/20
[52]	Nalidixic acid = 55/63, trimethoprim-sulfamethoxazole = 13/63, cephalothin = 12/63, ceftazidime = 7/63, colistin = 15/63, kanamycin = 16/63
[47]	Ampicillin = 19/19, kanamycin = 19/19, oxacillin = 19/19, spectinomycin = 19/19, streptomycin = 19/19 sulfamethoxazole/trimethoprim = 19/19
[71]	Ampicillin = 11/30, apramycin = 4/30, cefotaxime = 7/30, ciprofloxacin = 9/30, clindamycin = 30/30, colistin sulphate = 2/30, levofloxacin = 3/30, lincomycin = 30/30, nalidixic acid = 13/30, norfloxacin = 1/30, streptomycin = 11/30, tetracycline = 10/30 trimethoprim-sulfamethoxazole = 8/30
[72]	Clindamycin-158/158, oxacillin = 158/158, streptomycin = 154/158, tetracycline = 124/158, nalidixic acid = 112/158, amikacin = 90/158, sulphamethoxazole-trimethoprim = 86/158, ampicillin = 76/158, kanamycin = 68/158, cefotaxime = 62/158, colistin = 62/158, gentamicin = 48/158, ciprofloxacin = 44/158, chloramphenicol = 34/158, meropenem = 12/158, levofloxacin = 8/158
[73]	Colistin = 20/23, ampicillin = 15/23, doxycycline’s = 11/23
[109]	Amoxycillin-clavulanic acid = 114/115, cefazolin = 113/115, nalidixic acid = 89/115, cefoxitin = 89/115, tetracycline = 88/115, cefepime = 84/115
[44]	High resistance against ampicillin, streptomycin, oxacillin, tetracycline
[74]	Ampicillin = 65/100, trimethoprim-sulfamethoxazole = 67/100, cefoxitin = 68/100, ceftazidime = 78/100, tetracycline = 70/100, imipenem = 20/100, meropenem = 18/100, gentamicin = 0/100
[75]	Ampicillin = 18/40, Amoxicillin/clavulanic = 38/40, chloramphenicol = 24/40, tetracycline = 37/40, levofloxacin = 39/40, nalidixic acid = 40/40, trimethoprim = 20/40, nitrofurantoin = 32/40
[76]	Gentamicin = 2/11, amoxicillin = 4/11, trimethoprim/ sulphamethoxazole = 2/11, ampicillin = 11/11, doxycycline = 2/11, oxytetracycline = 5/11, ciprofloxacin = 3/11, cefpodoxime = 0/11
[110]	Nalidixic acid = 71/105, ciprofloxacin = 9/105, ampicillin = 10/105, Azithromycin = 31/105, tetracyclines = 66/105, trimethoprim-sulfamethoxazole = 44/105, chloramphenicol = 41/105
[77]	Penicillin, tylosin, tetracycline, erythromycin, tiamulin = 4/4, trimethoprim/sulfamethoxazole, difloxacin and lincomycin/ spectinomycin = 2/4, flumequine and florfenicol = 1/4
[111]	Chicken: Amikacin = 18/30, amoxicillin-clavulanic = 16/30, ampicillin = 14/30, ampicillin-sulbactam = 13/30, aztreonam = 21/30, cefotaxime = 24/30, ceftazidime = 16/30, ceftriaxone = 22/30, cephalexin = 8/30, ciprofloxacin = 20/30, gentamicin = 9/30, imipenem = 25/30, sulfamethoxazole-trimethoprim = 20/30; Human: Amoxicillin-clavulanic = 1/14, ampicillin = 2/14, ampicillin-sulbactam = 3/14, aztreonam = 2/14, cefotaxime = 2/14, ceftriaxone = 2/14, cephalexin = 1/14, ciprofloxacin = 1/14, gentamicin = 2/14, imipenem = 2/14
[78]	Erythromycin = 19/19, ampicillin = 19/19, colistin = 5/19
[112]	Chicken: Oxytetracycline = 6/15, ciprofloxacin = 3/15, cephalothin = 11/15, neomycin = 6/15, erythromycin = 15/15, nalidixic acid = 9/15, ampicillin = 1/15, doxycycline = 2/15, kanamycin = 6/15, streptomycin = 15/15, cefotaxime = 8/15, gentamicin = 5/15, amikacin = 5/15, sulphamethoxazol = 12/15, penicillin = 6/15 Humans: Oxytetracycline = 8/17, ciprofloxacin = 4/17, cephalothin = 9/17, neomycin = 7/17, erythromycin = 16/17, nalidixic acid = 13/17, ampicillin = 3/17, doxycycline = 6/17, kanamycin = 6/17, streptomycin = 17/17, cefotaxime = 8/17, gentamicin = 2/17, amikacin = 4/17, sulphamethoxazol = 10/17, penicillin = 11/17
[113]	Chloramphenicol = 9/9, ampicillin = 9/9, doxycycline = 8/9, cefepime = 4/9, ciprofloxacin = 1/9
[45]	Erythromycin = 10/10, vancomycin = 9/10, nalidixic acid = 9/10, ampicillin = 9/10, streptomycin = 6/10
[49]	Amoxicillin-clavulanic acid = 46/46, ceftriaxone = 39/46, sulfamethoxazole = 39/46, tetracycline = 36/46, cefotaxime = 37/46, Ceftazidime = 30/46, gentamicin = 27/46
[80]	Tetracycline = 4/27, gentamicin = 9/27, cefepime = 27/27, ciprofloxacin = 2/27
[81]	Ampicillin = 28/34, amoxicillin-clavulanic acid = 9/34, cefoxitin = 10/34, ceftiofur = 17/34, ceftriaxone = 7/34, nalidixic acid = 21/34, ciprofloxacin = 1/34, sulfisoxazole = 22/34, trimethoprim-sulfamethoxazole = 31/34, streptomycin = 14/34, gentamicin = 11/34, tetracycline = 31/39
[115]	Aminoglycosides, fluoroquinolones, aminocoumarins, sulfonamides, tetracyclines
[82]	Sulfamethoxazole–trimethoprim = 100%, amoxicillin = 100%, oxytetracycline = 100%, ceftazidime, cefotaxime = 85%, gentamycin, neomycin = 65%
[83]	Amoxicillin-clavulanic acid = 10/10, streptomycin = 10/10, erythromycin, clindamycin = 10/10, doxycycline = 10/10, fosfomycin = 10/10, sulfamethoxazole-trimethoprim = 10/10, ofloxacin = 9/10, apramycin = 8/10, ceftriaxone = 7/10, gentamycin = 7/10
[116]	Clindamycin = 87/87, streptomycin = 86/87, nalidixic acid = 79/87, penicillin = 66/87
[84]	Tetracycline = 15/16, Ampicillin = 11/16, ceftriaxone and cefoxitin = 9/16, Ciprofloxacin = 7/16
[85]	Tetracycline = 97.1%, nalidixic acid = 93.3%, ampicillin = 92.4%, azithromycin = 75.2%, ciprofloxacin = 63.8%, ceftriaxone = 54.3%
[50]	Ampicillin = 21/27, amoxicillin-clavulanic = 23/27, ceftazidime = 11/27, cefepime = 6/27, aztreonam = 14/27, amikacin = 23/27, gentamicin = 24/27, tobramycin = 27/27, ciprofloxacin = 20/27, nitrofurantoin = 8/27, trimethoprim/sulfamethoxazole = 9/27
[86]	Amoxicillin = 55/89, gentamicin = 29/89, sulfamethoxazole-trimethoprim = 71/89, doxycycline = 74/89, tetracycline = 74/89, ciprofloxacin = 81/89, cefotaxime = 34/89, nalidixic acid = 77/89, fosfomycin = 4/89, ampicillin = 68/89, ceftazidime = 7/89, chloramphenicol = 58/89
[88]	Meropenem = 13/129, trimethoprim/sulphamethoxazole = 40/129, nalidixic acid = 107/129, levofloxacin = 65/129, ciprofloxacin = 63/129, tetracycline = 65/129, gentamicin = 42/129, cephalothin = 55/129, cefaclor = 61/129, cefotaxime = 82/129, cefepime = 106/129, azithromycin = 15/129, fosfomycin = 37/129, ceftazidime/Clavulanic acid = 67/129, vancomycin = 119/129
[89]	Ampicillin = 10/10, cefpodoxime = 10/10, cefoxitin = 10/10, oxacillin = 10/10, cefotaxime = 8/10, ceftazidime = 7/10, ciprofloxacin = 6/10, ceftriaxone = 6/10, nalidixic acid = 6/10, Amoxicillin–clavulanic acid = 5/10
[90]	Cefpodoxime = 26/30, streptomycin = 16/30, tetracycline = 13/30, Trimethoprim/sulphamethoxazole = 25/30, ampicillin = 27/30, amoxycillin and clavulanic acid = 27/30, rifampicin = 18/30
[91]	Gentamicin = 0/83, ampicillin = 80/83, streptomycin = 65/83, neomycin = 30/83, cefotaxime = 5/83, colistin = 7/83, trimethoprim/sulfamethoxazole = 39/83, ciprofloxacin = 3/83, tetracycline = 56/83, florfenicol = 24/83, tiamulin = 81/83, flumequine = 63/83, enrofloxacin = 7/83
[92]	Tetracycline = 167/15, ampicillin = 159/185, amoxicillin = 136/185, chloramphenicol = 136/185, nalidixic acid = 132/185
[93]	Imipenem = 17/22, ciprofloxacin = 15/22, ceftazidime = 14/22, nalidixic acid = 13/22, ceftriaxone = 12/22
[94]	Ampicillin = 80/106, amoxicillin/clavulanic acid = 48/106, ceftazidime = 10/106, cefotaxime = 26/106, gentamicin = 8/106, chloramphenicol = 21/106, tetracycline = 65/106, imipenem = 12/106, sulfafurazole = 70/106, ciprofloxacin = 45/106
[96]	Nalidixic acid = 97/97, ampicillin = 81/97, amoxicillin–clavulanate = 78/97, ciprofloxacin = 78/97, tetracycline = 37/97, streptomycin = 12/97, chloramphenicol = 11/97
[46]	Amoxicillin-clavulanate = 32/166, ampicillin = 95/166, cefotaxime = 25/166, chloramphenicol = 51/166, ciprofloxacin = 57/166, nalidixic acid = 67/166, gentamicin = 38/166, tetracycline = 86/116, trimethoprim-sulfamethoxazole = 63/166, quinolones/fluoroquinolones = 64/166
[97]	Cefoxitin = 29/29, cefepime = 29/29, ceftazidime = 29/29, nalidixic acid = 29/29, erythromycin = 29/29, fosfomycin = 29/29, amoxicillin = 27/29, ampicillin = 27/29, aztreonam = 28/29, amoxicillin-clavulanic acid = 28/29, ceftriaxone = 26/29 chloramphenicol = 26/29, ciprofloxacin = 27/29, gentamicin = 23/29, streptomycin = 27/29, kanamycin = 27/29, apramycin = 22/29, doxycycline = 24/29, trimethoprim/ sulfamethoxazole = 28/29
[117]	Streptomycin = 19/19, erythromycin = 17/19, clindamycin = 14/19, ampicillin = 12/19, cefazolin = 11/19, tetracycline = 8/19
[98]	Cefoxitin, sulfisoxazole, and nalidixic acid exhibited the highest resistance rates
[99]	Ciprofloxacin = 25/37, nalidixic acid = 24/37, tetracyclines = 4/37
[118]	Gentamicin = 13/13, ciprofloxacin = 13/13
[101]	Spectinomycin = 25/25, tigecycline = 25/25, amoxicillin-clavulanic acid = 25/25, cefoxitin = 25/25, cefalexin = 25/25, cefazolin = 25/25, ampicillin = 23/25, cefotaxime = 25/25, sulphatrimethoprim = 20/25, chloramphenicol = 18/25, nalidixic acid = 15/25 colistin = 9/25, enrofloxacin = 10/25, ofloxacin = 5/25, ciprofloxacin = 5/25
[102]	Ampicillin = 6/19, cefotaxime = 2/19, ceftazidime = 7/19, cefpodoxime = 17/19, cefoxitin = 9/17, cefoperazone = 5/19, azithromycin = 7/19 tetracycline = 11/19, doxycycline = 4/19, ciprofloxacin, nalidixic acid = 14/19, trimethoprim sulfamethoxazole = 6/19, chloramphenicol = 7/19
[103]	Amoxicillin = 18/19, amoxicillin-clavulanic acid = 18/19, ceftriaxone = 16/19, ceftazidime = 19/19, cefepime = 19/19, chloramphenicol = 16/19, doxycycline = 16/19, ciprofloxacin = 17/19, erythromycin = 19/19, gentamicin = 17/19, streptomycin = 17/19
Clinical isolates	
[31]	High resistance to ampicillin
[30]	Amoxicillin = 20/20, ampicillin = 20/20, chloramphenicol = 20/20, lincomycin = 20/20, gentamicin = 20/20, nalidixic acid = 20/20, streptomycin = 20/20, trimethoprim = 20/20, neomycin = 18/20, norfloxacin = 18/20, tetracycline = 18/20, colistin sulphate = 2/20. ciprofloxacin = 4/20
[32]	Nalidixic acid = 93/202, tetracycline = 80/202, cotrimoxazole = 71/202, ampicillin = 28/202, chloramphenicol = 26/202, cefotaxime = 8/202, ceftriaxone = 8/202, ceftazidime = 8/202
[28]	Cefuroxime = 27/34, nalidixic acid = 16/34, ciprofloxacin = 15/34
[39]	Ampicillin = 11/138, ceftriaxone = 40/138, cefotaxim = 6/138, ceftazidime = 40/138
[35]	Nitrofurantoin = 34/200, Ampicillin = 27/200, cefalotin = 20/200, tigecycline= 20/200, ciprofloxacin = 17/200, trimethoprim/sulfamethoxazole = 16/200
[29]	Cefuroxime = 20/27, ciprofloxacin = 13/27
[33]	Nalidixic acid = 15/21, tetracycline (9/21), cotrimoxazole (6/21), ampicillin (6/21), chloramphenicol (6/21)
[38]	Nalidixic acid = 51/85
[37]	Ciprofloxacin = 12/15, piperacillin-tazobactam = 15/15, amikacin = 15/15, gentamicin = 15/15, levofloxacin = 11/15
[34]	Ciprofloxacin = 48/61, ampicillin = 6/10, gentamicin = 7/10
[36]	Tetracycline = 30/50, nalidixic acid = 50/50, cefazolin = 50/50, ampicillin = 30/50, chloramphenicol = 31/50, colistin = 8/50

**Table 5:** Subgroup analysis for the prevalence of *Salmonella* serovars across the GCC countries and ME region.

Subgroups	Studies	Estimate	LB	UP	SE	*p*-Value	I^2^ (%)
Country-wise							
Egypt	52	0.29	0.21	0.37	0.03	<0.001	99.72
Iran	19	0.35	0.25	0.46	0.05	<0.001	99.93
Iraq	5	0.10	0.05	0.14	0.02	<0.001	93.17
Jordan	3	0.40	−0.04	0.85	0.22	0.07	99.86
Kuwait	1	0.64	0.57	0.71	0.03	NA	NA
Kuwait and UAE	1	0.28	0.24	0.32	0.02	NA	NA
Lebanon	1	0.06	0.02	0.10	0.02	NA	NA
Palestine	1	0.03	0.01	0.04	0.007	NA	NA
Saudi Arabia	6	0.45	−0.02	0.93	0.24	0.06	99.97
UAE	4	0.14	0.03	0.25	0.05	0.01	98.65
Source-wise							
Food production environment							
Animal	66	0.31	0.24	0.38	0.03	<0.001	99.85
Both animal and human	9	0.27	0.18	0.35	0.04	<0.001	98.88
Human, animal and environment	1	0.12	0.09	0.16	0.01	NA	NA
Human and environment	1	0.33	0.23	0.42	0.04	NA	NA
Vegetables	1	0.01	0.002	0.02	0.006	NA	NA
Clinical isolates							
Human	15	0.28	0.06	0.50	0.11	0.01	99.98
Overall prevalence	93	0.30	0.23	0.36	0.03	<0.001	99.92

**Table 6:** Methodological quality assessment of the included studies.

Domain	Low Risk *n* (%)	Moderate Risk *n* (%)	High Risk *n* (%)
Sample representativeness	92 (97.87%)	1 (1.06%)	1 (1.06%)
Sample size adequacy	90 (95.74%)	2 (2.12%)	2 (2.12%)
Sampling methods	88 (93.61%)	4 (4.25%)	2 (2.12%)
Molecular methods	93 (98.93%)	1 (1.06%)	0 (0%)
Quality control	43 (45.74%)	0 (0%)	51 (54.25%)
Data analysis	44 (46.80%)	24 (25.53%)	26 (27.65%)
Reporting completeness	92 (97.87%)	2 (2.12%)	0 (0%)
Overall risk of bias	62 (65.95%)	1 (1.06%)	31 (32.97%)

**Table 7:** Certainty of evidence according to the GRADE framework for the assessment of main meta-analytic outcomes.

Outcome	Studies	Risk of Bias	Inconsistency	Indirectness	Imprecision	Publication Bias	Certainty Rating
Country and source-wise prevalence of Salmonella serovars	93	Low to high	Serious (I^2^ = 99.92%)	Serious	Not serious	Unclear	⊕ Very Low
Prevalence of ARGs in ME countries	15	Low	Serious (I^2^ = 96.95%)	Serious	Not serious	Unclear	⊕ Very Low
Prevalence of ARGs in food production environment and clinical isolates	15	Low	Serious (I^2^ = 97.4%)	Serious	Not serious	Unclear	⊕ Very Low
Prevalence of β-lactamase (country, source-wise, and variant-wise)	57	Low to high	Serious (I^2^ = 99.34%)	Serious	Not serious	Unclear	⊕ Very Low
Prevalence of tetracyclines (country, source-wise, and variant-wise)	30	Low	Serious (I^2^ = 99.62%)	Serious	Not serious	Unclear	⊕ Very low
Prevalence of aminoglycoside (country, source-wise, and variant-wise)	23	Low	Serious (I^2^ = 99.43%)	Serious	Not serious	Unclear	⊕ Very low
Prevalence of quinolone (country, source-wise, and variant-wise)	21	Low	Serious (I^2^ = 97.26%)	Serious	Not serious	Unclear	⊕ Very low
Prevalence of sulfonamide (country, source-wise, and variant-wise)	20	Low	Serious (I^2^ = 99.36%)	Serious	Not serious	Unclear	⊕ Very low
Prevalence of chloramphenicol (country, source-wise, and variant-wise)	8	Low	Serious (I^2^ = 99.79%)	Serious	Not serious	Unclear	⊕ Very low
MDR in ME countries	41	Low to high	Serious (I^2^ = 98.85%)	Serious	Not serious	Unclear	⊕ Very low

Abbreviations: ARGs = antibiotic resistance genes; MDR = multidrug-resistant.

## Data Availability

All data extracted from included studies are presented in the manuscript and Supplementary Materials.

## Supplementary Materials

The following supporting information can be downloaded at: https://www.mdpi.com/article/10.3390/life16091458/s1, Table S1: PRISMA 2020 Checklist. Table S2: Literature search strategy across databases. Table S3: Subgroup analysis for the prevalence of genotypic resistance patterns in GCC and ME countries. Table S4: Risk of bias assessments for individual studies.
