# Supplementary material for: Antibiotic Resistance Genes in Salmonella Isolates from Food Production Environments and Clinical Sources in the Middle East: A Systematic Review and Meta-Analysis Revealing the Limited Comparative Genomic Evidence
Source: Life (Basel). 2026 Aug 31;16(9):1458. doi: 10.3390/life16091458 (PMC13608501; doi:10.3390/life16091458)
Supplement: Supplementary file 1 [file life-16-01458-s001.zip › life-4483531-supplementary.pdf]

Table S1. PRISMA 2020 Checklist.

Section and Topic	Item #	Checklist item	Location where item is reported
TITLE			
Title	1	Identify the report as a systematic review.	Page 1
ABSTRACT			
Abstract	2	See the PRISMA 2020 for Abstracts checklist.	Page 1
INTRODUCTION			
Rationale	3	Describe the rationale for the review in the context of existing knowledge.	Page 2
Objectives	4	Provide an explicit statement of the objective(s) or question(s) the review addresses.	Page 2, 3
METHODS			
Eligibility criteria	5	Specify the inclusion and exclusion criteria for the review and how studies were grouped for the syntheses.	Page 3
Information sources	6	Specify all databases, registers, websites, organisations, reference lists and other sources searched or consulted to identify studies. Specify the date when each source was last searched or consulted.	Page 3
Search strategy	7	Present the full search strategies for all databases, registers and websites, including any filters and limits used.	Page 4, Supplementary Table 2
Selection process	8	Specify the methods used to decide whether a study met the inclusion criteria of the review, including how many reviewers screened each record and each report retrieved, whether they worked independently, and if applicable, details of automation tools used in the process.	Page 4
Data collection process	9	Specify the methods used to collect data from reports, including how many reviewers collected data from each report, whether they worked independently, any processes for obtaining or confirming data from study investigators, and if applicable, details of automation tools used in the process.	Page 4
Data items	10a	List and define all outcomes for which data were sought. Specify whether all results that were compatible with each outcome domain in each study were sought (e.g. for all measures, time points, analyses), and if not, the methods used to decide which results to collect.	Page 4, 5
	10b	List and define all other variables for which data were sought (e.g. participant and intervention characteristics, funding sources). Describe any assumptions made about any missing or unclear information.	Page 4, 5

 https://doi.org/10.3390/xxxxx

Section and Topic	Item #	Checklist item	Location where item is reported
Study risk of bias assessment	11	Specify the methods used to assess risk of bias in the included studies, including details of the tool(s) used, how many reviewers assessed each study and whether they worked independently, and if applicable, details of automation tools used in the process.	Page 5
Effect measures	12	Specify for each outcome the effect measure(s) (e.g. risk ratio, mean difference) used in the synthesis or presentation of results.	Page 5
Synthesis methods	13a	Describe the processes used to decide which studies were eligible for each synthesis (e.g. tabulating the study intervention characteristics and comparing against the planned groups for each synthesis (item #5)).	Page 5
	13b	Describe any methods required to prepare the data for presentation or synthesis, such as handling of missing summary statistics, or data conversions.	Page 5
	13c	Describe any methods used to tabulate or visually display results of individual studies and syntheses.	Page 5
	13d	Describe any methods used to synthesize results and provide a rationale for the choice(s). If meta-analysis was performed, describe the model(s), method(s) to identify the presence and extent of statistical heterogeneity, and software package(s) used.	Page 5
	13e	Describe any methods used to explore possible causes of heterogeneity among study results (e.g. subgroup analysis, meta-regression).	NA
	13f	Describe any sensitivity analyses conducted to assess robustness of the synthesized results.	NA
Reporting bias assessment	14	Describe any methods used to assess risk of bias due to missing results in a synthesis (arising from reporting biases).	NA
Certainty assessment	15	Describe any methods used to assess certainty (or confidence) in the body of evidence for an outcome.	Page 6
RESULTS			
Study selection	16a	Describe the results of the search and selection process, from the number of records identified in the search to the number of studies included in the review, ideally using a flow diagram.	Page 6
	16b	Cite studies that might appear to meet the inclusion criteria, but which were excluded, and explain why they were excluded.	Page 6 and Figure 1
Study characteristics	17	Cite each included study and present its characteristics.	Page 7-21; Table 1-3
Risk of bias in studies	18	Present assessments of risk of bias for each included study.	Page 36, 37, Table 7 and Supplementary Table 4

Section and Topic	Item #	Checklist item	Location where item is reported
Results of individual studies	19	For all outcomes, present, for each study: (a) summary statistics for each group (where appropriate) and (b) an effect estimate and its precision (e.g. confidence/credible interval), ideally using structured tables or plots.	Page 21-26, Table 4
Results of syntheses	20a	For each synthesis, briefly summarise the characteristics and risk of bias among contributing studies.	Page 7-26; Tables 1-4
	20b	Present results of all statistical syntheses conducted. If meta-analysis was done, present for each the summary estimate and its precision (e.g. confidence/credible interval) and measures of statistical heterogeneity. If comparing groups, describe the direction of the effect.	Page 27-36, Table 5, 6; Figure 2-4
	20c	Present results of all investigations of possible causes of heterogeneity among study results.	NA
	20d	Present results of all sensitivity analyses conducted to assess the robustness of the synthesized results.	NA
Reporting biases	21	Present assessments of risk of bias due to missing results (arising from reporting biases) for each synthesis assessed.	NA
Certainty of evidence	22	Present assessments of certainty (or confidence) in the body of evidence for each outcome assessed.	Page 33,34; Table 7
DISCUSSION			
Discussion	23a	Provide a general interpretation of the results in the context of other evidence.	Page 35-37
	23b	Discuss any limitations of the evidence included in the review.	Page 37-38
	23c	Discuss any limitations of the review processes used.	Page 37-38
	23d	Discuss implications of the results for practice, policy, and future research.	Page 37-38
OTHER INFORMATION			
Registration and protocol	24a	Provide registration information for the review, including register name and registration number, or state that the review was not registered.	Page 3
	24b	Indicate where the review protocol can be accessed, or state that a protocol was not prepared.	NA
	24c	Describe and explain any amendments to information provided at registration or in the protocol.	Page 3
Support	25	Describe sources of financial or non-financial support for the review, and the role of the funders or sponsors in the review.	Page 39
Competing interests	26	Declare any competing interests of review authors.	Page 39
Availability of data, code and other materials	27	Report which of the following are publicly available and where they can be found: template data collection forms; data extracted from included studies; data used for all analyses; analytic code; any other materials used in the review.	Page 39

From: Page MJ, McKenzie JE, Bossuyt PM, Boutron I, Hoffmann TC, Mulrow CD, et al. The PRISMA 2020 statement: an updated guideline for reporting systematic reviews. BMJ 2021;372:n71. doi: 10.1136/bmj.n71. This work is licensed under CC BY 4.0. To view a copy of this license, visit https://creativecommons.org/licenses/by/4.0/

Table S2. Literature search strategy across databases.

Database	Search terms (Free text and MeSH Terms)
PubMed	((("Salmonella"[MeSH Terms] OR "Salmonella enterica"[MeSH Terms] OR "antibiotic resistance" OR "antibiotic resistance bacteria" OR "antibiotic-resistant bacteria" OR "antimicrobial resistance" OR "AMR" OR "resistance gene" OR "antibiotic resistance gene" OR "ARG" OR "blaCTX-M" OR "blaCMY" OR "mcr" OR "tet gene")) AND (("food production" OR "livestock"[MeSH Terms] OR "poultry"[MeSH Terms] OR "farms"[MeSH Terms] OR "slaughterhouse" OR "food processing" OR "retail food" OR "clinical*" OR "hospital*" OR "patient*" OR "genetic similarity" OR "whole genome sequencing"[MeSH Terms] OR "WGS" OR "multilocus sequence typing"[MeSH Terms] OR "sequence type" OR "molecular typing"[MeSH Terms] OR "comparative genomics"))) AND (("Middle East" OR "Middle East Regions" OR "Middle East countries" OR "Gulf Cooperation Council countries" OR "GCC" OR "Gulf" OR "UAE" OR "Saudi Arabia" OR "Kuwait" OR "Qatar" OR "Bahrain" OR "Oman" OR "Iran" OR "Iraq" OR "Jordan" OR "Lebanon" Or "Egypt" OR "Yemen" OR "Syria")) Filters: Clinical Study, Clinical Trial, Clinical Trial, Phase I, Clinical Trial, Phase II, Clinical Trial, Phase III, Clinical Trial, Phase IV, Clinical Trial Protocol, Comparative Study, Controlled Clinical Trial, Evaluation Study, Evidence Synthesis, Multicenter Study, Observational Study, Randomized Controlled Trial, Randomized Controlled Trial, Veterinary, Validation Study, Clinical Trial, Veterinary, Observational Study, Veterinary
Scopus	TITLE-ABS-KEY (("Salmonella" OR "Salmonella enterica") AND ("antibiotic resistance" OR "antibiotic resistance bacteria" OR "antibiotic-resistant bacteria" OR "antimicrobial resistance" OR "AMR" OR "resistance gene" OR "antibiotic resistance gene" OR "ARG" OR "blaCTX-M" OR "blaCMY" OR "mcr" OR "tet gene") AND ("food production" OR "livestock" OR "poultry" OR "farms" OR "slaughterhouse" OR "food processing" OR "retail food" OR "clinical*" OR "hospital*" OR "patient*" OR "genetic similarity" OR "whole genome sequencing" OR "WGS" OR "multilocus sequence typing" OR "sequence type" OR "molecular typing" OR "comparative genomics") AND ("Middle East" OR "Middle East Regions" OR "Middle East countries" OR "Gulf Cooperation Council countries" OR "GCC" OR "Gulf" OR "UAE" OR "Saudi Arabia" OR "Kuwait" OR "Qatar" OR "Bahrain" OR "Oman" OR "Iran" OR "Iraq" OR "Jordan" OR "Lebanon" OR "Egypt" OR "Yemen" OR "Syria")) AND (LIMIT-TO (DOCTYPE , "ar")) AND (LIMIT-TO (LANGUAGE , "English") OR LIMIT-TO (LANGUAGE , "Arabic")) AND (LIMIT-TO (AFFILCOUNTRY , "Egypt") OR LIMIT-TO (AFFILCOUNTRY , "Saudi Arabia") OR LIMIT-TO (AFFILCOUNTRY , "Iraq") OR LIMIT-TO (AFFILCOUNTRY , "Kuwait") OR LIMIT-TO (AFFILCOUNTRY , "Lebanon") OR LIMIT-TO (AFFILCOUNTRY , "United Arab Emirates") OR LIMIT-TO (AFFILCOUNTRY , "Jordan") OR LIMIT-TO (AFFILCOUNTRY , "Qatar") OR LIMIT-TO (AFFILCOUNTRY , "Oman") OR LIMIT-TO (AFFILCOUNTRY , "Yemen") OR LIMIT-TO (AFFILCOUNTRY , "Bahrain") OR LIMIT-TO (AFFILCOUNTRY , "Morocco") OR LIMIT-TO (AFFILCOUNTRY , "Palestine")) AND (LIMIT-TO (SUBJAREA , "MEDI") OR LIMIT-TO (SUBJAREA , "IMMU") OR LIMIT-TO (SUBJAREA , "VETE") OR LIMIT-TO (SUBJAREA , "AGRI") OR LIMIT-TO (SUBJAREA , "BIOC") OR LIMIT-TO (SUBJAREA , "PHAR") OR LIMIT-TO (SUBJAREA , "ENVI") OR LIMIT-TO (SUBJAREA , "MULT") OR LIMIT-TO (SUBJAREA , "SOCI") OR LIMIT-TO (SUBJAREA , "CENG") OR LIMIT-TO (SUBJAREA , "HEAL") OR LIMIT-TO (SUBJAREA , "NURS"))

Web of Science	TS= (("Salmonella" OR "Salmonella enterica") AND ("antibiotic resistance" OR "antibiotic resistance bacteria" OR "antibiotic-resistant bacteria" OR "antimicrobial resistance" OR "AMR" OR "resistance gene" OR "antibiotic resistance gene" OR "ARG" OR "blaCTX-M" OR "blaCMY" OR "mcr" OR "tet gene") AND ("food production" OR "livestock" OR "poultry" OR "farms" OR "slaughterhouse" OR "food processing" OR "retail food" OR "clinical*" OR "hospital*" OR "patient*" OR "genetic similarity" OR "whole genome sequencing" OR "WGS" OR "multilocus sequence typing" OR "sequence type" OR "molecular typing" OR "comparative genomics") AND ("Middle East" OR "Middle East Regions" OR "Middle East countries" OR "Gulf Cooperation Council countries" OR "GCC" OR "Gulf" OR "UAE" OR "Saudi Arabia" OR "Kuwait" OR "Qatar" OR "Bahrain" OR "Oman" OR "Iran" OR "Iraq" OR "Jordan" OR "Lebanon" OR "Egypt" OR "Yemen" OR "Syria"))
ScienceDirect	Title, abstract, keywords: (“Salmonella”) AND (“antibiotic resistance gene”) AND (“food production” OR “livestock” OR “poultry” OR “clinical” OR “hospital” OR “patient”)
Google Scholar	(“Salmonella”) AND (“antibiotic resistance gene”) AND (“food production” OR “livestock” OR “poultry” OR “clinical” OR “hospital” OR “patient”)

Table S3. Subgroup analysis for the prevalence of genotypic resistance patterns in GCC and ME countries.

Subgroups	Studies	Estimate	LB	UP	SE	P-value	I^2 (%)
β-lactamase							
Country-wise							
Egypt	27	0.55	0.39	0.71	0.08	<0.001	98.83
Iran	14	0.35	0.17	0.52	0.09	<0.001	99.1
Iraq	2	0.69	0.17	1.22	0.26	0.009	94.4
Jordan	7	0.33	0.09	0.58	0.12	0.007	99.57
Saudi Arabia	3	0.56	0.42	0.76	0.27	0.04	99.6
UAE	2	0.59	0.42	0.76	0.08	<0.001	0%
Kuwait and UAE	1	0.12	0.06	0.10	0.03	NA	NA
Qatar	1	0.86	0.69	1.03	0.08	NA	NA
Source-wise							
Food production environment							
Animal	44	0.50	0.37	0.63	0.06	<0.001	99.34
Animal and human	3	0.48	0.02	0.93	0.23	0.03	98.13
Animal, human and environment	1	0.42	0.19	0.64	0.11	NA	NA
Clinical isolates							
Humans	9	0.36	0.21	0.52	0.08	<0.001	98.59
Variant-wise							
blaTEM	27	0.64	0.52	0.77	0.06	<0.001	98.51
blaSHV	7	0.32	0.09	0.56	0.12	0.006	99.27
blaCTXM	11	0.32	0.20	0.44	0.06	<0.001	96.98
blaOXA	4	0.26	0.009	0.51	0.12	0.04	97.08
blaNDM	2	0.08	-0.04	0.20	0.06	0.19	73.68
blaCMY	4	0.47	-0.05	1.00	0.27	0.07	99.5
blaDHA	2	0.39	-0.23	1.02	0.32	0.22	97.61
Overall prevalence	57	0.48	0.37	0.58	0.05	<0.001	99.34
Tetracyclines							
Country-wise							
Egypt	9	0.75	0.60	0.90	0.07	<0.001	97.33
Iran	9	0.42	0.16	0.68	0.13	0.001	99.02
Iraq	2	0.63	0.14	1.13	0.25	0.01	91.22
Jordan	7	0.37	0.16	0.58	0.10	<0.001	99.52
UAE	3	0.32	-0.04	0.68	0.18	0.08	97.73
Source-wise							

Food production environment							
Animal	26	0.51	0.33	0.69	0.09	<0.001	99.67
Animal and human	4	0.47	0.23	0.72	0.12	<0.001	90.57
Variant-wise							
tetA	17	0.65	0.52	0.79	0.06	<0.001	98.27
tetB	6	0.34	0.01	0.66	0.16	0.03	99.12
tetC	2	0.55	-0.26	1.36	0.41	0.18	99.83
tetK	1	0.28	0.20	0.35	0.03	NA	NA
tetD	2	0.32	-0.20	0.85	0.26	0.22	99.02
tetG	1	0.01	-0.002	0.03	0.009	NA	NA
tetE	1	0.17	0.12	0.23	0.02	NA	NA
Overall prevalence	30	0.51	0.34	0.67	0.08	<0.001	99.62
Aminoglycoside							
Country-wise							
Egypt	10	0.57	0.28	0.85	0.14	<0.001	99.37
Iran	4	0.41	0.09	0.72	0.15	0.01	98.37
Iraq	2	0.27	0.08	0.45	0.09	0.004	0
Jordan	4	0.31	0.01	0.61	0.15	0.04	99.26
Qatar	1	0.96	0.88	1.05	0.04	NA	NA
Saudi Arabia	1	0.76	0.70	0.81	0.02	NA	NA
UAE	1	0.91	0.69	1.13	0.11	NA	NA
Food production environment							
Animal	14	0.43	0.26	0.60	0.08	<0.001	98.67
Animal and human	7	0.55	0.19	0.90	0.18	0.002	99.56
Vegetables	1	0.91	0.69	1.13	0.11	NA	NA
Clinical isolates							
Humans	1	0.96	0.88	1.05	0.04	NA	NA
Variant-wise							
aadA1	7	0.61	0.41	0.0	0.10	<0.001	96.98
aadA2	2	0.99	0.97	1.01	0.009	<0.001	0
aadB	1	0.06	0.01	0.11	0.02	NA	NA
aadC	1	0.95	0.83	1.07	0.06	NA	NA
aac(6′)-Ib-cr	1	0.23	0.08	0.38	0.07	NA	NA
aac(3)- IV	2	0.19	0.05	0.32	0.07	0.007	0
strA	2	0.14	0.09	0.19	0.02	<0.001	7.23

strA/B	2	0.32	-0.03	0.67	0.18	0.07	97.88
strB	1	0.10	0.05	0.14	0.02	NA	NA
aac-(3)-Iia`11	1	0.21	0.15	0.27	0.03	NA	NA
aac(6′)-Iaa	2	0.96	0.88	1.04	0.04	<0.001	0
aac(3)	1	0.76	0.70	0.81	0.02	NA	NA
Overall prevalence	23	0.51	0.33	0.69	0.09	<0.001	99.43
Quinolone							
Country-wise							
Egypt	10	0.40	0.18	0.62	0.11	<0.001	97.47
Iran	5	0.47	0.07	0.87	0.20	0.02	97.66
UAE	4	0.29	0.06	0.51	0.11	0.01	93.6
Iraq	1	0.12	0.02	0.23	0.05	NA	NA
Qatar	1	0.80	0.59	1.00	0.10	NA	NA
Source-wise							
Food production environment							
Animal	14	0.30	0.18	0.43	0.06	<0.001	94.34
Animal and humans	1	0.96	0.86	1.06	0.05	NA	NA
Clinical isolates							
Humans	6	0.52	0.18	0.87	0.17	0.003	97.17
Variant-wise							
qnrA	5	0.37	0.00	0.75	0.19	0.05	98.31
qnrB	7	0.18	0.10	0.27	0.04	<0.001	68.88
qnrS	7	0.50	0.24	0.76	0.13	<0.001	96.6
gyrA	1	0.96	0.88	1.05	0.04	NA	NA
qnrB19	1	0.57	0.40	0.73	0.08	NA	NA
Overall prevalence	21	0.40	0.25	0.55	0.07	<0.001	97.26
Sulfonamide							
Country-wise							
Egypt	7	0.72	0.48	0.95	0.12	<0.001	99.54
Iran	7	0.50	0.29	0.72	0.11	<0.001	97.82
Iraq	1	0.57	0.47	0.67	0.05	NA	NA
Qatar	2	0.91	0.80	1.01	0.05	<0.001	0
UAE	3	0.34	-0.09	0.78	0.22	0.12	98.55
Source-wise							
Food production environment							

Animal	14	0.52	0.31	0.74	0.10	<0.001	99.53
Animal and human	2	0.73	0.24	1.22	0.25	0.003	94.64
Clinical isolate							
Humans	4	0.88	0.77	0.99	0.05	<0.001	30.58
Variant-wise							
sul1	12	0.65	0.46	0.84	0.09	<0.001	99.17
sul2	6	0.47	0.17	0.77	0.15	0.002	98.42
sul3	2	0.64	-0.01	1.30	0.33	0.05	99.66
Overall prevalence	20	0.60	0.43	0.77	0.08	<0.001	99.36
Chloramphenicol							
Country-wise							
Egypt	4	0.85	0.69	1.01	0.08	<0.001	92.02
Iran	2	0.98	0.96	1.01	0.01	<0.001	0
Jordan	2	0.35	-0.26	0.97	0.31	0.26	99.65
Source-wise							
Food production environment							
Animal	6	0.74	0.35	1.13	0.19	<0.001	99.85
Animal and human	2	0.78	0.63	0.92	0.07	<0.001	46.02
Variant-wise							
floR	5	0.81	0.65	0.97	0.08	<0.001	94.97
cat1	3	0.67	0.02	1.31	0.33	0.04	99.93
Overall prevalence	8	0.76	0.43	1.09	0.16	<0.001	99.79

Table S4. Risk of bias assessments for individual studies. 65

Study ID	Domains							Overall risk of bias
	Sample representativeness	Sample size adequacy	Sampling methods	Molecular methods	Quality control	Data analysis	Reporting	
[53]	Low	Low	Low	Low	Low	Moderate	Low	Low
[40]	Low	Low	Low	Low	Low	Moderate	Low	Low
[54]	Low	Low	Low	Low	Low	Low	Low	Low
[55]	Low	Low	Low	Low	High	High	Low	High
[56]	Low	Low	Low	Low	Low	Low	Low	Low
[57]	Low	Low	Low	Low	High	High	Low	High
[58]	Low	Low	Low	Low	High	High	Low	High
[59]	Low	Low	Low	Low	Low	Low	Low	Low
[60]	Low	Low	Low	Low	Low	Moderate	Low	Low
[41]	Low	Low	Low	Low	Low	High	Low	Low
[61]	Low	High	High	Low	High	High	Low	High
[42]	Low	Low	Low	Low	High	Moderate	Low	High
[62]	Low	Low	Low	Low	High	Low	Low	Low
[63]	Low	Low	Low	Low	Low	Low	Low	Low
[64]	Low	Low	Low	Low	Low	Low	Low	Low
[43]	Low	Low	Low	Low	High	Low	Low	Low
[65]	Low	Moderate	Moderate	Low	Low	Moderate	Low	Moderate
[66]	Low	Low	Low	Low	High	Low	Low	Low
[67]	Low	Low	Low	Low	High	Moderate	Low	High
[68]	Low	Low	Low	Low	High	High	Low	High
[69]	Low	Low	High	Low	High	High	Low	High
[70]	Low	Low	Low	Low	High	High	Low	High
[52]	Low	Low	Low	Low	Low	Low	Low	Low
[47]	Low	Low	Low	Low	Low	High	Low	Low

[71]	Low	Low	Low	Low	High	Moderate	Low	High
[72]	Low	Low	Low	Low	High	High	Low	High
[73]	Low	Low	Low	Low	High	Low	Low	Low
[109]	Low	Low	Low	Low	Low	Moderate	Low	Low
[74]	Low	Low	Low	Low	Low	Low	Low	Low
[75]	Low	Low	Low	Low	High	Low	Low	Low
[76]	Low	Low	Low	Low	High	High	Low	High
[51]	High	High	Moderate	Low	Low	High	Moderate	High
[77]	Low	Low	Low	Low	High	Moderate	Low	High
[78]	Low	Low	Low	Low	Low	Low	Low	Low
[79]	Low	Low	Low	Low	High	Low	Low	Low
[45]	Low	Low	Low	Low	High	Moderate	Low	High
[80]	Low	Low	Low	Low	Low	Low	Low	Low
[81]	Low	Low	Low	Low	Low	Moderate	Low	Low
[82]	Low	Low	Low	Low	High	Low	Low	Low
[83]	Low	Low	Low	Low	High	Low	Low	Low
[84]	Low	Low	Low	Low	Low	Low	Low	Low
[85]	Low	Low	Low	Low	Low	Low	Low	Low
[86]	Low	Low	Low	Low	High	Low	Low	Low
[87]	Moderate	Moderate	Moderate	Low	High	Moderate	Low	High
[88]	Low	Low	Low	Low	High	Low	Low	Low
[89]	Low	Low	Low	Low	High	High	Low	High
[90]	Low	Low	Low	Low	High	Low	Low	Low
[91]	Low	Low	Low	Low	Low	Low	Low	Low
[92]	Low	Low	Low	Moderate	High	High	Low	High
[93]	Low	Low	Low	Low	Low	Low	Low	Low
[94]	Low	Low	Low	Low	High	Low	Low	Low
[95]	Low	Low	Low	Low	Low	High	Low	Low
[96]	Low	Low	Low	Low	Low	Low	Low	Low
[46]	Low	Low	Low	Low	High	Low	Low	Low

[97]	Low	Low	Low	Low	High	Low	Low	Low
[98]	Low	Low	Low	Low	Low	Low	Low	Low
[99]	Low	Low	Low	Low	Low	Moderate	Low	Low
[100]	Low	Low	Low	Low	Low	Low	Low	Low
[101]	Low	Low	Low	Low	High	Low	Low	Low
[102]	Low	Low	Low	Low	High	Low	Low	Low
[103]	Low	Low	Low	Low	High	Low	Low	Low
[104]	Low	Low	Low	Low	High	Moderate	Low	High
[105]	Low	Low	Low	Low	Low	High	Low	Low
[106]	Low	Low	Low	Low	Low	High	Low	Low
[107]	Low	Low	Low	Low	Low	Low	Low	Low
[108]	Low	Low	Low	Low	Low	High	Low	Low
[109]	Low	Low	Low	Low	Low	High	Moderate	High
[110]	Low	Low	Low	Low	Low	Low	Low	Low
[111]	Low	Low	Low	Low	High	Moderate	Low	High
[112]	Low	Low	Low	Low	High	Low	Low	Low
[113]	Low	Low	Low	Low	High	Low	Low	Low
[114]	Low	Low	Low	Low	Low	Moderate	Low	Low
[115]	Low	Low	Low	Low	High	Low	Low	Low
[116]	Low	Low	Low	Low	High	Moderate	Low	High
[117]	Low	Low	Low	Low	High	Moderate	Low	High
[118]	Low	Low	Low	Low	Low	Moderate	Low	Low
[119]	Low	Low	Moderate	Low	High	Moderate	Low	High
[120]	Low	Low	Low	Low	High	High	Low	High
[121]	Low	Low	Low	Low	High	Low	Low	Low
[48]	Low	Low	Low	Low	Low	Low	Low	Low
[49]	Low	Low	Low	Low	Low	Low	Low	Low
[50]	Low	Low	Low	Low	Low	Moderate	Low	Low
[29]	Low	Low	Low	Low	Low	High	Low	Low
[31]	Low	Low	Low	Low	High	Moderate	Low	High
[30]	Low	Low	Low	Low	Low	Low	Low	Low
[32]	Low	Low	Low	Low	Low	High	Low	Low

[28]	Low	Low	Low	Low	High	High	Low	High
[39]	Low	Low	Low	Low	High	High	Low	High
[35]	Low	Low	Low	Low	High	High	Low	High
[33]	Low	Low	Low	Low	High	High	Low	High
[38]	Low	Low	Low	Low	High	Moderate	Low	High
[37]	Low	Low	Low	Low	High	Low	Low	Low
[34]	Low	Low	Low	Low	Low	Moderate	Low	Low
[36]	Low	Low	Low	Low	Low	Low	Low	Low
